# Revisiting the mechanism of coagulation factor XIII activation and regulation from a structure/functional perspective

**DOI:** 10.1038/srep30105

**Published:** 2016-07-25

**Authors:** Sneha Gupta, Arijit Biswas, Mohammad Suhail Akhter, Christoph Krettler, Christoph Reinhart, Johannes Dodt, Andreas Reuter, Helen Philippou, Vytautas Ivaskevicius, Johannes Oldenburg

**Affiliations:** 1 Institute of Experimental Haematology and Transfusion Medicine, University Clinic Bonn, 53127 Bonn, Germany; 2 Department of Molecular Membrane Biology, Max Planck institute of Biophysics, 60439 Frankfurt, Germany; 3 Paul Ehrlich Institute, 63225 Langen, Germany; 4 Leeds Institute for Cardiovascular and Metabolic Medicine, University of Leeds, Leeds, UK

## Abstract

The activation and regulation of coagulation Factor XIII (FXIII) protein has been the subject of active research for the past three decades. Although discrete evidence exists on various aspects of FXIII activation and regulation a combinatorial structure/functional view in this regard is lacking. In this study, we present results of a structure/function study of the functional chain of events for FXIII. Our study shows how subtle chronological submolecular changes within calcium binding sites can bring about the detailed transformation of the zymogenic FXIII to its activated form especially in the context of FXIIIA and FXIIIB subunit interactions. We demonstrate what aspects of FXIII are important for the stabilization (first calcium binding site) of its zymogenic form and the possible modes of deactivation (thrombin mediated secondary cleavage) of the activated form. Our study for the first time provides a structural outlook of the FXIIIA_2_B_2_ heterotetramer assembly, its association and dissociation. The FXIIIB subunits regulatory role in the overall process has also been elaborated upon. In summary, this study provides detailed structural insight into the mechanisms of FXIII activation and regulation that can be used as a template for the development of future highly specific therapeutic inhibitors targeting FXIII in pathological conditions like thrombosis.

The fibrin stabilizing factor or coagulation factor XIII (FXIII) is a heterotetrameric protein complex, circulating in the plasma as a 320 KDa molecule consisting of a dimer of A subunits (FXIIIA_2_, 83 kDa) and a dimer of B subunits (FXIIIB_2_, 80 kDa)^1^,^2^,^3^. FXIII belongs to the transglutaminase family of enzymes (EC 2.3.2.13)^4^. The catalytic FXIIIA_2_ subunit possesses transglutaminase activity that covalently crosslinks fibrin polymers to confer resistance against premature fibrinolysis^5^. Deficiency of FXIII can result in a bleeding predisposition from acquired or inherited causes^6^,^7^. Patients with severe inherited FXIII deficiency (complete absence or loss of function) suffer from severe bleeding tendencies. Inherited severe FXIII deficiency is a rare autosomal disorder with a global average of approximately 1–4 out of a million individuals being affected^6^. However, a more frequently inherited form of FXIII deficiency is the heterozygous form that is usually associated with a mild or even asymptomatic phenotype^6^,^7^,^8^,^9^,^10^,^11^. This form is difficult to detect and, therefore is currently under-reported and poorly characterized. The past few decades of research have shown FXIII to have multiple pleiotropic functions^12^. It is known to play roles in maintaining vascular permeability, in development of extracellular matrix in bone and cartilage, in fostering cardioprotective effects, as a first line of defense against invading pathogens and, as recently reported, in pre-adipocyte differentiation and arthritis^13^,^14^,^15^,^16^,^17^,^18^. Identification and cloning of the *F13A1* and *F13B* genes, along with the recombinant expression of the FXIIIA_2_ and B_2_ subunits^19^,^20^,^21^, was followed by high-resolution structural determination of the FXIIIA_2_ dimer in zymogenic forms^22^,^23^,^24^,^25^. Recently the structure of calcium-activated and inhibitor-stabilized FXIIIA subunit (FXIIIAa) was solved which shows remarkable differences from the zymogenic form^26^. Structurally, the FXIIIA subunit is composed of four sequentially arranged structural units: the beta sandwich, core, barrel-1 and barrel-2 domains. Unique to the FXIIIA subunit in the transglutaminase family is the presence of a 37 amino acid N-terminal activation peptide (FXIII-AP) which is cleaved by thrombin during FXIII activation. Despite early achievements investigating secondary structural elements and structural domains of FXIIIB^21^,^26^, progress in structure/function studies of this non-catalytic subunit have been slow and there are no high-resolution X-ray crystallographic or NMR structures for FXIIIB monomers, putative dimers, or for the FXIIIA_2_-bound conformation in FXIIIA_2_B_2_ tetramer. High primary sequence homologies with proteins from the complement system suggest that the monomeric FXIIIB subunit is composed of ten Sushi domains, each comprising ~60 amino acid residues^21^,^27^,^28^. Limited information exists on the interaction between FXIIIA and FXIIIB subunits and on FXIIIB_2_ dimerization^29^,^30^. The FXIIIB subunit is known to have a protective role, although more recently a regulatory role has also come to light^31^,^32^. The FXIIIA subunit has been a potential target for developing therapeutic inhibitors against thrombotic conditions. Earlier, the primary targeted region for developing therapeutic inhibitors was the thrombin cleavage site and the catalytic triad in the zymogenic form of the FXIIIA subunit^33^,^34^,^35^,^36^,^37^,^38^. With emerging details of structural changes taking place during FXIII activation, the activated form (FXIIIAa) is being considered as a good potential target^26^,^38^. The activation of the FXIIIA_2_B_2_ heterotetramer in plasma is an elaborate process involving the cleavage of FXIII-AP by thrombin combined with calcium binding which causes large-scale conformational changes in the FXIIIA subunit structure and also results in the dissociation of the FXIIIB subunits^26^,^39^. Although a number of studies by various groups have shed light on different aspects of this complicated process, however a comprehensive understanding of the complete activation process is lacking. In the current study we present a structure/function study of the chain of events comprising FXIII heterotetramer dissociation, FXIIIA subunit activation and subsequent down-regulation. We use computational analysis to elucidate structure/function associations that relate various stages of the entire FXIII pathway. Complimentary to this, we performed gel filtration analysis to characterize the dissociation of the FXIIIA_2_B_2_ heterotetrameric complex in different local biochemical conditions. In addition, we have tried to offer an alternative explanation for the regulatory role of FXIIIB subunit on FXIIIA activation *in vitro*. Finally, we compare observations and conclusions from the present study with results and conclusions derived from past literature.

## Materials and Methods

### Simulation and comparison of FXIIIA_2_ and Transglutaminase-2 structures

In order to understand the inter-residue relationships within the different parts of FXIIIA subunit and tissue transglutaminase-2 (TG2) plain molecular dynamics (MD) simulation was performed on the zymogenic human FXIIIA_2_ crystal structure (PDB ID: 1f13; 2.1 Å resolution)^24^ and the zymogenic TG2 structure (PDB ID: 1kv3; *H. Sapiens* species; 2.1 Å resolution)^40^ using the YASARA Structure package version 13.11.1^41^,^42^. Gaps or unresolved regions within the crystal structure(s) were modeled them on the FREAD loop modeling server (http://opig.stats.ox.ac.uk/webapps/fread/php/; accessed on 05.10.2014)^43^. For e.g. the PDB file 1f13 that is unresolved at the thrombin cleavage site Arg37-Gly38 was submitted to the server under default parameters. The final structurally resolved structure was chosen based on scores that were a combination of all backbone atom anchor match RMSD(corresponds to the base structure) and all backbone atom loop match RMSD (corresponds to the loop structure). The PDB files were initially subjected to 500 ps of refinement MD simulation using YAMBER3 force field parameters in YASARA in order to remove steric clashes and improve rotamer geometry^41^. The file with the lowest energy in the simulation trajectory was chosen for conducting further simulations. In order to simulate the structures, a simulation cell with periodic boundaries and 20 Å minimum distances to protein atoms was employed with explicit solvent. The AMBER03 force field, NPT ensemble was used with long range PME potential and a cut-off of 7.86 Å^44^. Hydrogen bond networks were optimized using the method of Hooft and co-workers^45^. The simulation cell was filled with water at a density of 0.997 g/mL and a maximum sum of all bumps per water of 1.0 Å. The simulation cell net charge was neutralized with a final 0.9% (wt/vol) NaCl concentration. The entire system was energy minimized by steepest descent to remove conformation stress within the structure, followed by simulated annealing minimization until convergence was achieved. The MD simulation was performed at three different temperatures i.e. 298 K, 340 K and 370 K. Simulations for both structures were run for 100 ns (nanoseconds). Structural image visualization, analysis and rendering were done with YASARA 13.11.1 and Chimera version 1.10.2^42^,^46^. Solvation energies for initial crystal structures as well as for simulation trajectory snapshots were calculated by submitting trajectory converted PDB-formatted files to the PDBePISA server (http://www.ebi.ac.uk/pdbe/pisa/pistart.html; accessed on 05.01.2015)^47^. Electrostatic surface potential was calculated and graphically depicted using the Adaptive Poisson-Boltzmann Solver integrated within YASARA^48^. The extent of correlated motion calculated in YASARA for all simulations is represented by C(i, j). It is collected in matrix form and displayed as a three-dimensional dynamic cross-correlation map (DCCM). The time scale is implicit in the C (i, j) values. The cross correlation was calculated as average over the time period of the entire trajectory. Positively correlated residues move in the same direction, whereas (negatively) anti-correlated residues move in the opposite direction. A completely correlated or anti-correlated motion, C (i, j) = 1 or C (i, j) = −1, means that the motions have the same phase and period. Blue color represent value 1 and yellow −1, all intermediate values are depicted by shades which are semi-proportionate mix of these two colors. The color code is represented as an inset diagram for all matrices.

### Removal of bound calcium from zymogenic FXIII crystal structures using steered molecular dynamic simulation

Steered molecular dynamics (SMD) simulation was performed on the monomer (A chain) of the calcium-bound (at Ala457, Cab1) human FXIIIA subunit zymogenic structure (PDB ID: 1 ggu; 2.1 Å resolution)^25^ to simulate relocation of bound Ca^2+^ ions into the bulk solvent. The PDB file was passed through a refinement simulation protocol (described before) to generate an energy minimized structure before conducting the SMD^41^. The SMD simulation was conducted in two steps: 1) An initial 10 ns classical MD simulation with parameters as described above (for files 1f13 and 1kv3) followed by 2) a steered simulation during the production phase in which steering potentials were applied on the complex. After 10 ns of classical MD run, a steering force with an acceleration of 1000 pm/ps^2^ (picometre/picosecond square) was imposed on the bound ligand Calcium. The direction of the force was applied from the geometric center of the receptor to the geometric center of the ligand. The steered simulation was halted and analyzed at the point at which the Ca^2+^ ion was displaced into and completely surrounded by hydrated counter ions. No part of the whole system was restrained during the production phase SMD or the initial MD. Upon reaching the simulation end point, the resulting protein models and the simulation averaged structures were compared to the starting crystallographic structures and scored for backbone alpha-carbon (or side chain atom) Root mean squared displacement (RMSD). The simulation trajectory was also analyzed for variations in RMSD and Root mean square fluctuations (RMSF). A plain MD simulation with parameters similar to those described for 1f13 and 1kv3 in the earlier section was also run with two PDB files: 1) the simulation refined 1 ggu file with calcium bound at the first calcium binding site 2) a simulation refined 1 ggu file but with the calcium manually removed from its first calcium binding site. These simulations were run for 100 ns each at 298 K, 340 K and 370 K and the simulation end point structures and simulation averaged structures were compared with each other by structural alignment (results shown in main images correspond only to simulation run at 298 K).

### Steered molecular dynamic simulation of zymogenic FXIII structures with and without the activation peptide

A similar SMD methodology to the one applied for calcium removal from zymogenic FXIIIA was employed for simulating dissociation of individual monomers from two separate homodimeric FXIIIA_2_ structures: one a zymogenic human Factor XIIIA_2_ structure including the activation peptide (PDB ID: 1f13; 2.1 Å resolution) and the other with N-terminal FXIII-AP residues through Pro39 deleted. Like before both structures were initially minimized using a structure refinement protocol^41^. A steering force with an acceleration of 100 pm/ps^2^ was then applied to displace monomer chain B from monomer chain A. No part of the whole system was restrained during the SMD or the initial MD to allow freedom of movement for residues being influenced by the separation of the monomers. The SMD was analyzed to the point at which no intermolecular contacts were observed between the FXIIIA monomers and/or the atoms started crossing the periodic boundaries.

### Modeling all-atom factor XIII activation pathway intermediates

To generate FXIII activation pathway intermediate structural models, we used two crystal structures of FXIIIA_2_: the starting zymogenic FXIIIA_2_ crystal structure (PDB ID: 1f13; 2.1 Å resolution) and the non-proteolytically (high Ca^2+^ concentration) activated FXIIIAa crystal structure (PDB ID: 4kty; 1.8 Å resolution)^24^,^26^. The intermediates were generated by submitting these two structures as end-state structures to the ANMPathway server (http://anmpathway.lcrc.anl.gov/anmpathway.cgi; accessed on 08.11.2014), which uses a coarse-grained modeling approach to construct a two-state potential calculated by combining two elastic network models (ENMs) representative of the experimental structures representing the beginning and end points of the simulation^49^. Intermediate structures are extracted as snapshots along continuous steepest descent pathways generated for the protein atomic coordinates during the transition from beginning to end point structures. Since the server requires the beginning and end states to be similar in terms of molecular content, all atoms and molecules not common to both input structural coordinate files were deleted, missing/unresolved residues were modeled by modeling them (as described before), and only monomeric chains from each file were submitted. The AMNPathway server replaces amino acid side-chain atoms with a single representative vdW shell and returns alpha-carbon backbone traces without explicit side chain atoms as results. Once a large number of coarse grained intermediates were generated, eight were finally selected which differed from each other by at least >2 Å RMSD (of the alpha carbons of the backbone trace). The side chains of these intermediates were modeled using the PD2 ca2main server (http://www.sbg.bio.ic.ac.uk/~phyre2/PD2_ca2main; accessed on 25.11.2014) to generate the corresponding full atom models^50^. Full atom models were then subjected to a structure refinement protocol as described before^41^. Lowest energy models were selected from the simulation trajectory and analyzed. All the transition intermediate as well as end state models have been deposited in the protein modeling database (https://bioinformatics.cineca.it/PMDB/main.php) (PMDBID: PM0080129-PM0080136).

### An all-atoms partial heterotetrameric model of FXIIIA_2_B_2_

In order to generate a partial model of the FXIIIA_2_B_2_ heterotetramer, we used a step-wise approach to first generate a dimeric modeled structure of FXIIIB_2_, followed by a constrained docking of a limited number of contiguous Sushi domains on the zymogenic FXIIIA_2_ subunit crystal structure. A threaded model of a monomeric FXIIIB subunit was at first generated on the I-TASSER server (http://zhanglab.ccmb.med.umich.edu/I-TASSER/; accessed on 11.12.2014) with distance constraints applied for the known disulfide bonded cysteine’s within each Sushi domain of the FXIIIB subunit^51^. Since the auto generated structure had certain flaws i.e. absence of specific disulfide bond, presence of disordered structures in ordered (predicted) areas etc., all ten individual sushi domains of this threaded model were replaced with previously modeled structures of these domains while keeping the linker regions of the threaded monomeric FXIIIB subunit model^28^. The linker region of the automated structure was not touched in order to retain the backbone properties of this earlier model. The modified monomeric FXIIIB model was subjected to a round of model refinement simulation protocol as described before and the final energetic minimum monomeric structure was chosen from the trajectory^41^. The modified monomeric FXIIIB model was symmetrically dimerized using the M-ZDOCK docking server (http://zlab.umassmed.edu/m-zdock/; accessed on 12.01.2015), to generate a dimeric structure^52^. The ZDOCK server searches the translational and rotational space between the two proteins for all possible binding modes and evaluates and ranks each pose using an energy-based scoring function. The M-ZDOCK is an adapted from the ZDOCKing algorithm to predict structures of symmetric or cyclically symmetric multimers. The server generated (based on its scores) top ten docking poses, only one of which showed a head to tail (antiparallel) orientation with close proximity between the Sushi domains 4 and 9 on adjacent chains (with oppositely charged electrostatic patches) and this was chosen as the final model for the FXIIIB_2_ structure (based on current experimental data; see Supplementary methods for more details)^29^,^53^. The docking pose was energy minimized with a 100 ns long classical MD simulation with parameters as described before i.e. simulation of PDB files 1f13 and 1kv3 (trajectory details i.e. RMSD/energy values can be made available on request). The lowest energy conformers from these simulations were subjected to a model refining simulation protocol as described earlier^41^. The lowest energy structure from this simulation trajectory was chosen as the final FXIIIB_2_ subunit dimeric model. In the absence of adequate experimental data to enable co-operative docking of the FXIIIIB_2_ subunit Sushi domains on the FXIIIA_2_ subunit, we performed blind docking of only a symmetrically identical end component of the FXIIIB_2_ dimer model (consisting of Sushi domains 1, 2, 3 and 4 of one monomer and Sushi domains 8, 9 and 10 of the opposite monomer) onto a monomeric chain of FXIIIA_2_ zymogenic crystal structure using the Z-dock server (http://zdock.umassmed.edu/; accessed on 21.01.2015). The model with closest proximity between Sushi domains 1 and 2 (as per what is currently known for FXIIIA: FXIIIB subunit interactions) and the monomeric FXIIIA was chosen as the final model. This model was subjected to rounds of energy minimizing classical MD simulation followed by refining simulation protocol as applied for the B_2_ subunit. The lowest energy structure from the final refinement protocol was once again chosen as the final model. The docked FXIIIA-FXIIIB (S1–S4 antiparallel S8–S10) model was then converted into a partial heterotetramer by dimerizing it using the M-ZDOCK server as described above. In this specific docking, all known non-interface residues (identified from the PDB file: 1f13 using PDBePISA) of the FXIIIA2 dimer were used as a negative constraint. This gave us a final partial heterotetrameric model of FXIIIA2B2 (S1–S4 antiparallel S8–S10). This final model was not subjected to any more rounds of classical MD simulation but minimized directly by refining it with the previously described 500 ps refinement protocol. However, in order to test for the stability of this docked complex it was subjected to 100 ns of classical MD simulation post the refinement simulation, following parameters described for the simulation of PDB files 1f13 and 1kv3. The primary FXIIIA_2_B_2_ heterotetrameric partial model has been deposited in the protein modeling database (https://bioinformatics.cineca.it/PMDB/main.php) (PMDBID: PM0080128).

### Dissociation of FXIIIB (S1–S4 antiparallel S8–S10) subunit component from the partial FXIIIAB heterodimer by steered molecular dynamic simulation

A similar methodology to the one applied for dissociating bound calcium from the zymogenic FXIIIA molecule was employed for dissociating the FXIIIB partial subunit component from the previously modeled partial FXIIIAB heterodimer [*i.e.*, consisting of one half of the previously generated partial heterotetrameric FXIIIA_2_B_2_ (S1–S4 antiparallel S8–S10) model]. A steering force with an acceleration of 200 pm/ps^2^ was applied on the FXIIIB subunit component. No part of the whole system was restrained during the SMD or the initial MD to allow freedom of movement for residues being influenced by the separation of the individual subunits. The steered simulation was analyzed to the point at which no intermolecular contacts were observed between the individual subunits and/or the atoms started crossing the periodic boundaries.

### Gel filtration analysis of the activation of FXIII A_2_B_2_ heterotetramer

Plasma concentrate *Fibrogammin P* (CSL Behring), was used a source of human Factor XIII heterotetramer. FXIII was purified using gel filtration chromatography on a Superdex 200 10/300 GL column (GE Healthcare). Briefly, the column was equilibrated with running buffer (30 mM Tris, 150 mM NaCl, pH 7.4), *Fibrogammin P* was loaded and fractions were eluted. Fractions corresponding to the molecular weight of FXIII heterotetramer (~320 kDa), were collected and sequentially re-purified thrice until a single, homogenous, monodispersed peak was observed. For each set of *in vitro* activation experiments, 25 μg of purified FXIIIA_2_B_2_ dissolved in 20 mM Tris, 120 mM NaCl, pH 7.4 buffer was incubated with 46.2 U/mL of thrombin (Sigma, USA), at different concentrations of calcium chloride (0 mM, 1 mM, 2 mM, 5 mM, 10 mM and 25 mM), for 60 minutes at 30 degrees. Reaction product was filtered and resolved using a Superdex 200 PC 3.2/30 (GE Healthcare) analytical column. Peaks from samples loaded at different calcium ion concentrations were collected and analyzed on Native PAGE (Life technologies, Germany). The observed bands were analyzed and confirmed for the presence of FXIIIA and FXIIIB by Mass Spectrometry (see Supplementary Data).

### Spiking FXIIIB during activated FXIIIA (FXIIIAa) generation

FXIIIAa generation was triggered by tissue factor/phospholipids (TF/PL) and FXIIIa isopeptidase activity was measured using the fluorogenic substrate A101 (Zedira, Darmstadt, Germany) in a Safire microtiter plate reader (Tecan, Craislheim, Germany)^54^. Twenty five microliters human standard plasma (Siemens Healthcare, Marburg, Germany) or FXIII-deficient plasma (deficient for FXIIIA_2_ and FXIIIB_2_; Haemochrom Diagnostica GmbH, Essen, Germany) spiked with rFXIII-A_2_ (1 IU/mL) or a mixture FXIIIA_2_ (1 IU/mL) (Purified *in-house*, see Supplementary data) and rFXIIIB_2_ (10 μg/mL) (Zedira, Darmstadt, Germany) were incubated with 35 μL reagent solution (5 μL 100 mM glycine methyl ester, 5 μL 2 mM fluorogenic FXIIIa substrate, 10 μL Innovin (recombinant TF; Dade Behring, Liederbach, Germany) diluted 1:2800 in phospholipids (PTT reagent kit, Roche, Mannheim, Germany) and 15 μL HBS (20 mM Hepes, 150 mM NaCl)/0.1% serum albumin pH 7.5. After pre-incubation of the mixture for 5 minutes, the reaction was started with 40 μL 25 mM CaCl_2_ pH 7.5. Fluorescence was measured over 1 h at λ_ex_ = 330 nm and λ_em_ = 430 nm in kinetic mode 2 times per minute. The curve data was evaluated according to a bi-exponential model with first order absorption and elimination. Data were fitted to the equation:


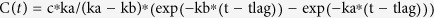


where ka – constant of absorption which describes the development of FXIIIa and kb – elimination constant. The parameters area under the curve (AUC), peak FXIIIa concentration (CP), and time to peak (TTP) were also evaluated.

## Results

### Simulation and comparison of FXIIIA_2_ and TG2 models: Structure/sequence differences between FXIIIA2 and TG2 account for their respective functional evolution

The FXIIIA (monomeric chain A) and TG2 zymogenic structures show perfect alignment of the respective domains except for the absence of the FXIII-AP region in TG2 (Figure S1). TG2 is known to bind 6 calcium ions of which 5 non-canonical sites have been characterized by mutagenesis^13^. The monomeric chain of the FXIIIA subunit possesses only 3 calcium binding sites (Cab1–3), but with similar spatial correlation to homologous TG2 sites. The RMSD graphs during the simulation run for both these structures at different temperatures are shown in Figure S1. The simulation for both FXIIIA2 and TG2 PDB files stabilized around ~2–2.5 Å RMSD after 10 and 40 ns respectively (total energy of the systems ~−37 × 10^6 ^kJ/mol and −29 × 10^6 ^kJ/mol respectively). For the FXIIIA2 and TG2 structures we observed a positive correlation (DCCM) between the residues of the separate calcium binding sites (TG2 mean C (*i*, *j*) = 0.462; FXIIIA_2_ mean C (*i*, *j*) = 0.186) (Figure S3). The TG2 redox switch^14^, which consists of two vicinal disulfide bonded cysteine’s, lies in a highly conserved region of FXIIIA_2_, although one of the cysteine’s is substituted by an arginine in FXIIIA_2_ which precludes the possibility of formation of an allosteric disulfide bond in FXIIIA_2_. The GTP/GDP binding site^15^ (Arg478/Ser482) of TG2 is not conserved in the FXIIIA_2_ dimer (Figure S1). Instead, the region aligns with a sequence of the FXIIIA subunit that forms a secondary thrombin cleavage site (Figure S2, Lys513/Ser514)^5^.

### A constitutively bound calcium at Cab1 stabilizes the zymogenic form of FXIIIA_2_

**Figure 1:**
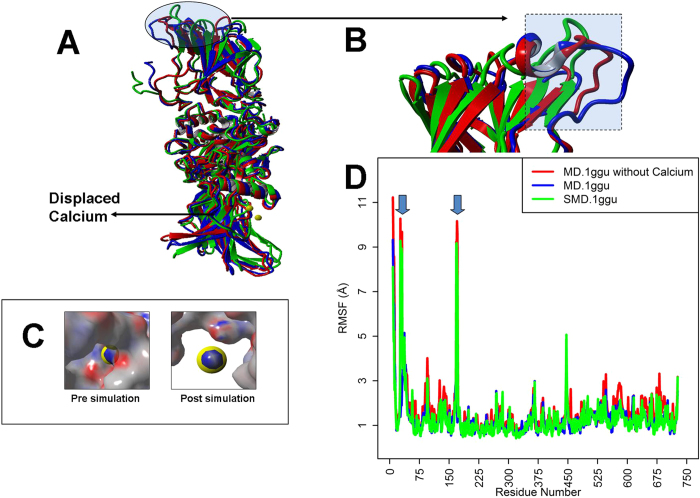
Steered Molecular Dynamic (SMD) simulation to dissociate bound calcium from FXIIIA models. (Panel **A**) The structural alignment of three simulation snapshots (one simulation end point structure for the SMD performed on 1 ggu colored blue; one a post 100 ns simulation snapshot of the 1 ggu file deprived of calcium at Cab1 colored red; one a post 100 ns simulation snapshot of the original 1 ggu file colored green) from one monomer (chain A) of the zymogenic FXIIIA_2_ structure (PDB ID: 1 ggu). Backbone structures are depicted in ribbons. Bound calcium is represented as a yellow ball. Shaded regions show maximum amount of displacement in secondary structure alpha-carbon backbone (>2 Å RMSD). (Panel **B**) Close up view of the structural alignment depicted in (Panel **A**) focusing on the region around the FXIII-AP cleavage site. Ribbon colors as for (Panel **A**). (Panel **C**) Molecular surface view of the calcium binding site pre- (left side) and post-SMD (right side) simulation. Red, negative potential; blue, positive potential; calcium ion, yellow ball. The electrostatic surface potential was calculated using YASARA. (Panel **D**) A RMSF(Root mean square fluctuation) graph comparing the average RMSF per residue for the plain simulation run on the PDB file 1 ggu with and without calcium and also of the SMD run on 1 ggu. Spikes in RMSF exclusively for the SMD run on 1 ggu and 1 ggu file without bound calcium are shown with downward pointing arrows.

Applying simulated dissociative force (using SMD) on calcium to remove it from the constitutively bound site at Cab1 of FXIIIA results in significant change of structure (>2 Å RMSD) around the FXIII-AP cleavage site (Fig. 1). Prior to MD simulation to displace bound calcium, this region was part of an ordered beta sheet (the regions between residue numbers 27–31 and 168–172 constitute two antiparallel beta strands stabilized by means of 4 hydrogen bonds). During simulation, these 4 hydrogen bonds that stabilize the sheet were lost and the antiparallel beta strands assumed a disordered random coils conformation for most of the simulation time. Interestingly, this region is spatially distant (>10 Å) from the calcium binding site, therefore the observed modeled structural change is most likely consistent with an allosterically mediated calcium binding effect. The plain MD simulation of calcium bound 1 ggu file did not show any remarkable differences in secondary structure post 100 ns of simulation in this region around the thrombin cleavage site [the Fig. 1 Panel A clearly shows that even post 100 ns of simulation this region is represented by a beta sheet (green colored)]. However when we simulated the 1 ggu PDB file after manually removing the bound calcium for 100 ns, we observed the loss of ordered secondary structure (see Fig. 1 Panel A; the red and blue backbone structures corresponding to this region in the shaded areas are disordered) in the region around the cleavage site as observed during the SMD in which calcium was pulled out. Unusually high RMSF values were observed in simulation averaged structures of the plain MD run for 1 ggu without calcium as well as for the SMD, but none for the plain MD run for 1 ggu with bound calcium (Fig. 1 Panel D; spikes 1 and 2; results correspond to simulations done at 298 K only although similar observations were registered for 340 and 370 K as well). The RMSD graphs for plain simulations run on the PDB files of 1 ggu with and without calcium are depicted in Figures S4. These figures show that both structures are relatively stable with or without calcium, although the PDB file without calcium achieves its RMSD plateau ~0.2 Å higher than with calcium which might be attributed to RMSD changes occurring around the thrombin cleavage site. Structural alignment of the simulation averaged structures from both simulations shows little overall structural difference (average RMSD: 1.342 Å). However a significantly higher average RMSD of 5.6 Å was observed between the thrombin cleavage site and neighboring residues (considering the P1–P4 and P1′–P4′ residues) of the two simulation averaged structures. This indicates local changes in structure (and hence of accessibility) around the thrombin cleavage site sessile bond following the removal of calcium.

### The activation peptide is the major contributor to the dimeric interface of the zymogenic Factor XIIIA_2_, and its cleavage weakens the dimer interaction

**Figure 2:**
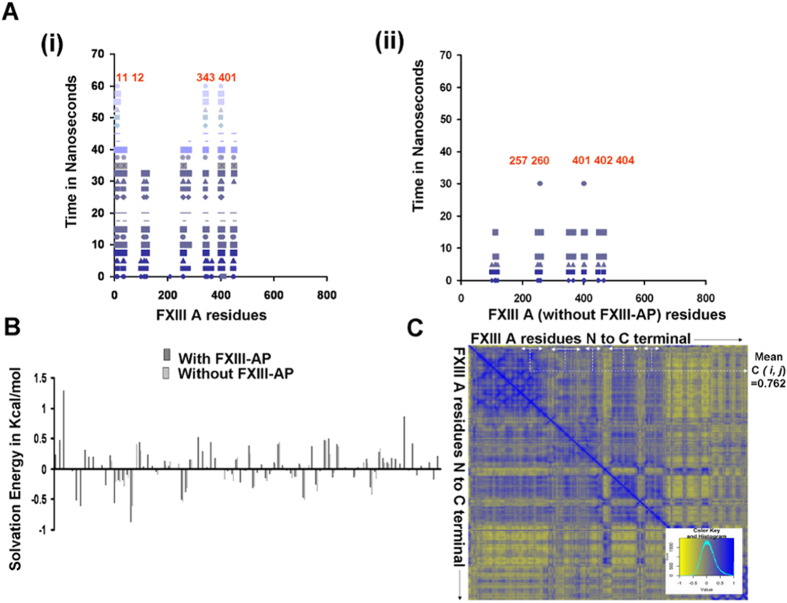
Steered molecular dynamic simulation of FXIIIA2 dimer dissociation into separated monomers. (Panel **A**) Separation plots showing physical interactions (each spot represents an interaction observed in a specific snapshot; the spots are colored in different shades of blue and spots from the same simulation snapshot have the same shade) for residues N to C terminal (x-axis) of one FXIIIA monomer at the dimeric interface over the progress time (y-axis) of the SMD (in picoseconds). (i) Interactions for the zymogenic FXIIIA structure (with FXIII-AP); (ii) interactions for the zymogenic structure without FXIII-AP (residue numbering for both plots according to the complete zymogen primary sequence). Tha main interacting partners in FXIIIA monomer, forming the dimeric interfaces in the both the cases, are labelled in red. (Panel **B**) Post-simulation mean solvation energies (y-axis) for the FXIIIA subunit dimeric interface residues (x-axis). Black bars, zymogenic FXIIIA_2_ with FXIII-AP, Grey, without FXIII-AP. (Panel **C**) Calculated DCCM (dynamic cross correlation matrix) values for the 60 ns SMD simulation of FXIIIA monomer dissociation from zymogenic FXIIIA_2_ dimer with FXIII-AP. Color key shown as inset: yellow, negative correlation; blue, positive correlation. The bidirectional white arrows represent the positively correlated region between the FXIII-AP residues and the corresponding FXIIIA dimeric interface residues (not including the FXIII-AP).

The SMD simulation to dissociate the individual monomers of FXIIIA_**2**_, with and without the FXIII-AP sequence, revealed that the structure without FXIII-AP becomes dissociated in less time (30 ns) at a constant separation force than the dimer with the intact activation peptides (60 ns) (Fig. 2, Panel A). The absence of FXIII-AP also results in a weaker interaction between non-FXIII-AP residues within the dimeric interface. The mean solvation energies of the residues on these interfaces (estimated from the simulations) are lower, exemplified by greater negative calculated values for the structure without FXIII-AP (Fig. 2, Panel B). Also, the residues that become separated by the end of the simulation are responsible for FXIII-AP/core domain interactions as judged by comparison with the starting crystallographic structure. The activation peptide residues show positive correlation [mean C (*i*, *j*) = 0.762] with respect to the rest of the dimeric interface residues (during the SMD of the zymogenic FXIIIA with FXIII-AP) (Fig. 2, Panel C). We observe that the interaction of two neighboring Arg residues on FXIII-AP, Arg11 and Arg12 with Asp343 and Glu401, respectively, on the core domain of the apposing monomer may be critical to the dimeric stability of the FXIIIA subunit structure since as observed, these interactions were last to be separated during simulation of subunit dissociation. The mean correlation value for these four residues with the rest of the dimeric interface is negative [mean C (*i*, *j*) = −0.647] indicating that they are displaced from their starting positions in a manner consistent with opposition to the dissociative forces experienced during the overall simulation.

### MD simulation suggests large conformational changes upon activation of FXIIIA is affected by calcium binding at three sites in a temporal manner

**Figure 3:**
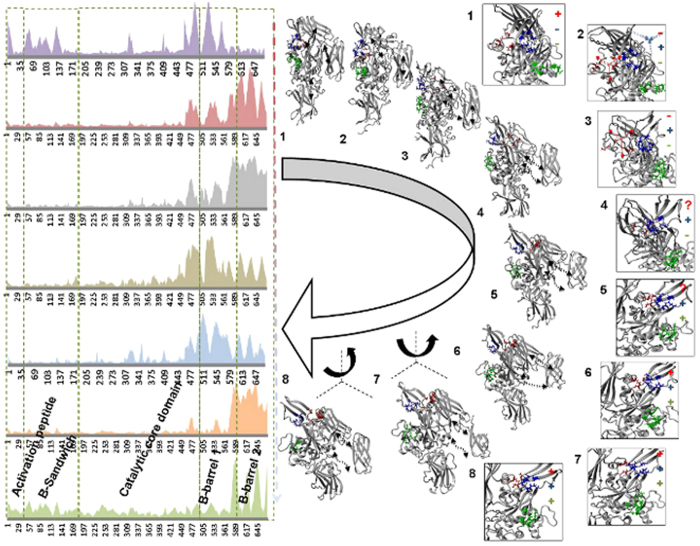
Domain displacements and structural changes in calcium binding sites for the simulated transition state intermediate models. Domain displacements and structural changes in calcium binding sites observed within transition state intermediate models (n = 8). RMSD values per backbone alpha-carbon for each residue of FXIIIA (left side). Y-axis values are normalized RMSD in Å, x-axis denotes the backbone alpha Carbon (N to C terminal), with domains specified. Next to arced open arrow (middle) are models of the 8 transition state intermediate (grey). Calcium binding sites are Cab1 (red), Cab2 (blue) and Cab3 (green). Direction of motions of domains due to calcium binding is shown with small black dashed double-headed arrows. The direction and relative magnitude of twisting motions are depicted next to intermediates 6–8 (straight dashed lines indicate x-, y- and z-axes for an arbitrary reference frame representing the orientation of each intermediate as shown). The boxed images (right) are close up views showing the location, formation and/or disruption of three calcium binding sites for the 8 intermediates. The coordination states of the calcium binding sites are indicated either with bound calcium (+) or with no calcium bound (−). Arrows indicate simulated movements of Ca^2+^ ions relative to each binding site. Residues forming the calcium binding sites are shown in stick format.

**Figure 4:**
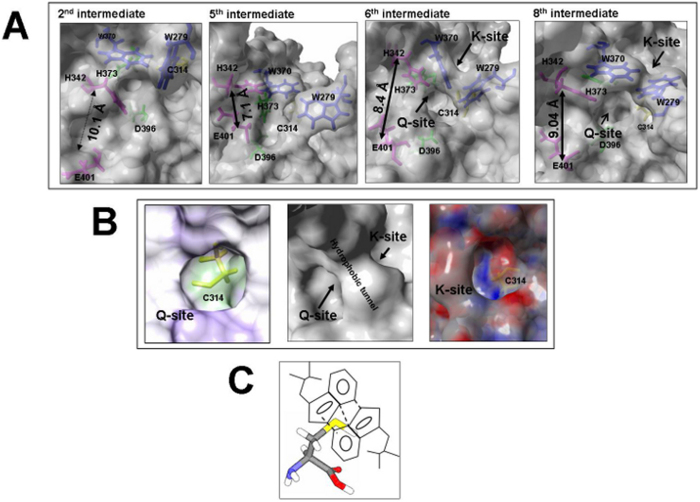
Detailed views of transition state intermediate models. (Panel **A**) Molecular surface representations of transition intermediate models 2, 5, 6 and 8 (left to right, respectively). The molecular surfaces (grey); key residues including catalytic triad (green), substrate guiding diads (magenta), and aromatic residues lining the hydrophobic tunnel (blue) shown as stick representations. (Panel **B**) Views of the FXIIIA subunit catalytic site in its fully activated (FXIIIAa) form as molecular surface representations (grey surfaces). Shown are entry of vestibule for the Q substrate binding site (i) relative hydrophobicity: minimum (purple) to maximum (green)), hydrophobic tunnel (ii), and entry point of vestiblue for the K substrate binding site (iii; electrostatic surface potentials: negative (red), positive (blue)); catalytic C314 (panels i and iii, yellow stick representation); molecular schematic representation of pi-stacking interactions (dashed lines) that stabilize the hydrophobic tunnel (iv). The electrostatic surface potential was calculated with YASARA. (Panel **C**) This is cartoonist illustration of the hydrophobic tunnel formed by the planar interaction of the Trp rings. The reactive cysteine is shown as a stick model at the bottom with sulphur atom colored yellow.

The end state models of the activation path intermediates show overall similarity in secondary structure although the complete backbone moves to a large extent (Figure S5). Comparison of models in the series suggests major changes in secondary structure in the region between residues 450 and 650 (intermediates 2 through 5) which is affected by local changes in calcium binding at Cab1 and Cab2 (Fig. 3). Changes in backbone structure during the simulation are initially observed around the beta sandwich region and parts of core and barrel-1 domains (Fig. 3, RMSD graphs). Structural changes shift to core domain, barrel-1 and barrel-2 in successive intermediates and translate to and/or rotate the two barrel domains with respect to each other and with respect to the core domain. Finally, backbone conformational changes appear to alternate between the barrel-1 and barrel-2 domains during the course of the transition, which results in movement of the barrel domains across their common plane of symmetry, thereby twisting the final molecule three dimensionally while causing the FXIIIA subunit to unfold like a jackknife (Fig. 3). During the simulation, the first calcium binding site (Cab1) becomes disrupted as the second calcium binding site (Cab2) is unmasked and coordinates a Ca^2+^ ion. The distance between Glu401 and His342 residues (referred as catalytic diad), which are distant in the zymogenic form, decreases through the early intermediates 1–4 (Fig. 4, Panel A, sections i, ii). This simulated movement appears to be a consequence of intermittent switching between beta strand and coil forms for the backbones of both Glu401 and His342 those results in a flapping movement of these residues close and then subsequently away from each other. A hydrophobic pocket, whose base is occupied by the catalytic Cys314 residue, emerges along with a hydrophobic tunnel formed by the movement of the planar rings Trp370 and Trp279 (also as a result of Cab2 coordination), which are finally stabilized by aromatic stacking interactions (Fig. 4, Panels A, B). On one side of this tunnel is the K substrate (Lysine substrate) entrance site and the other side is the His342/Glu401 diad known to guide the Q substrate (Glutamine substrate) to the base of the hydrophobic pocket where a positively charged oxyanion hole, formed by electronegative backbone carbonyl groups of both Cys314 and Trp279, is located. This oxyanion hole stabilizes the substrate-enzyme intermediate (Fig. 4, Panel B).

### The all-atoms partial heterotetrameric model of FXIIIA_2_B (S1–S4 S8–S10)_2_ suggests that bound FXIIIB subunits mediate regulation of FXIII activation

**Figure 5:**
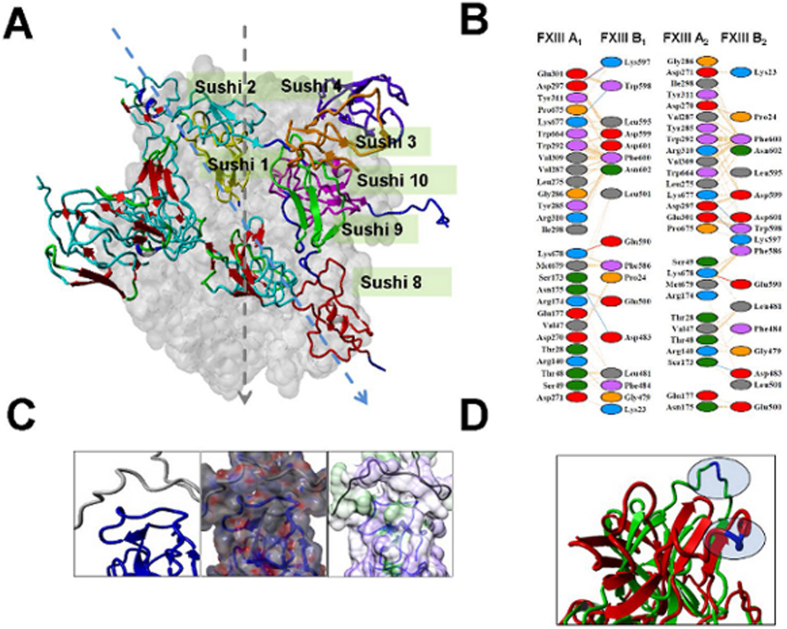
All-atoms partial heterotetrameric FXIIIIA2B (S1–S4 S8–S10)_2_ model. (Panel **A**) Heterotetrameric FXIIIIA_2_B (S1–S4 antiparallel S8–S10)_2_ model. FXIIIA subunit shown as molecular surface rendering (grey); Sushi domains from apposed FXIIIB monomers shown in alpha-carbon trace (ribbons) format. (colored according to secondary structure: beta-strand (red), extended loop (cyan), random coil (green), alpha-helix (blue); dashed arrow marks the mirror symmetry of both FXIIIA (grey) and FXIIIB (cyan) subunits. (Panel **B**) Schematic diagram of putative interface residues forming physical contacts between the FXIIIA and FXIIIB subunits of the heterotetrameric FXIIIIA_2_B (S1–S4 antiparallel S8–S10)_2_ model. The dashed lines represent inter-residue contacts while the multiple colored oval structures represent the participating residues. (Panel **C**) FXIIIB S1 Sushi domain interaction with the FXIII-AP N-terminal region. Backbone alpha-carbon trace (left panel, ribbons: S1 domain, blue; FXIII-AP, grey). Middle panel shows electrostatic potential (negative, red; positive, blue) superimposed on molecular surface view for same domains depicted in left panel. The electrostatic surface potential was calculated and graphically depicted using Adaptive Poisson-Boltzmann Solver (integrated within YASARA). Right panel shows hydrophobicity (minimum, purple; maximum, green) for the same molecular view as in left and middle panels. (Panel **D**) Close up view of aligned models from simulation snapshots. Pre- (green) and post-SMD (red) alpha-carbon backbone traces (ribbons) from 80 ns simulation of FXIIIB dissociation from FXIIIA in the vicinity of FXIII-AP.

A flowchart for the generation of the FXIIIA_2_B partial heterotetrameric model is presented in Figure S6. The FXIIIB_2_ subunit dimeric model is symmetric, long and filamentous as has been suggested from electron microscopy based studies (approximately 21.5 nm end to end)^27^. The first two N terminal Glu residues for this model were skipped from the final structure owing to conformational equilibrium purposes. This model had regions around sushi domains 4 and 9 interacting with each other on the opposite chains in a head to toe manner and therefore the overall structure was symmetrical. The regions around sushi domains 4 and 9 were observed to have oppositely charged surface electrostatic potential (Figure S7) which adequately fitted into each other. The final all-atoms partial heterotetrameric model was a symmetric fitting of the B subunit fragments around the A subunit. While the A subunit retained its former non-associated structure, the B subunit (fragments) appears to undergoe significant rearrangement to wrap around one side of the A subunit. This partial model for the FXIIIA_**2**_B_**2**_ complex was quite stable as can be seen from the plain simulation runs performed at 298 K in which it hits a equilibrated RMSD plateau around the 2.5 Å mark (c-α backbone RMSD)(Figure S8). Plain simulations performed at 370 K showed significantly higher RMSDs approaching the 3.5 and 4.5 Å respectively mark at 100 ns of runtime which is not unusual for a non covalently associated complex plus this model is lacking in possible binding contributory domains. The all-atoms partial heterotetrameric model of FXIIIA_**2**_B_**2**_ (Fig. 5, Panel A) suggests an interaction between the variable length loop region of FXIIIB Sushi domain 1 (Residues Tyr18-Pro28) with the N-terminal residues (Thr6-Asp25) of the FXIIIA subunit activation peptide (Fig. 5, Panel C). The interaction appears to be primarily hydrophobic since although the activation peptide region bears a strong positive electrostatic potential, the variable length loop of Sushi domain 1 is electrostatically neutral. Other regions of interaction are defined in Fig. 5, Panel B. In this model, the Cab1 site is always exposed and therefore occupied by a calcium ion, Cab2 is occluded by the FXIIIB S1/FXIII-AP interaction surface, while the third Cab3 is partially occluded by the FXIIIA_2_ dimeric interface, but, more significantly, by the FXIIIB_**2**_ subunit Sushi 1/Sushi 2 domains from apposing monomers.

### Intermediate partial FXIIIA_2_B (S1–S4 S8–S10)_2_ complex models generated during SMD simulated activation suggest successive weakening of intersubunit interactions

**Figure 6:**
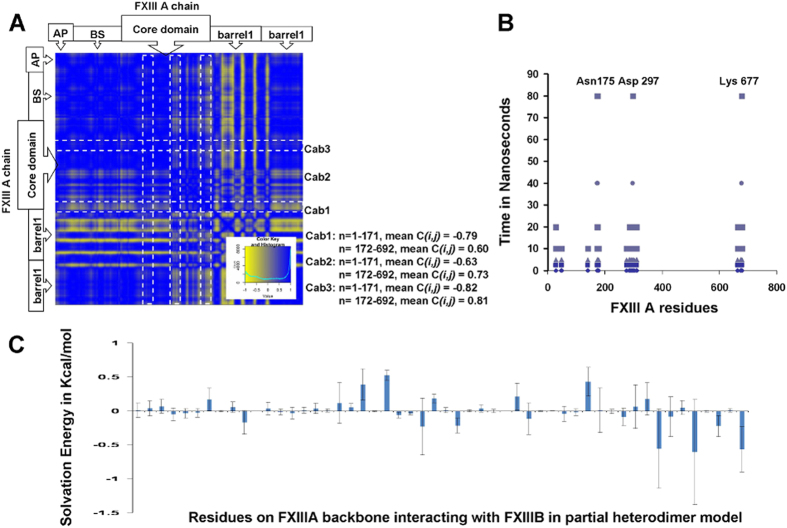
SMD simulation results for FXIIIB subunit dissociation from FXIIIA subunit. (Panel **A**) Inter-residue displacement (DCCM) correlation calculated for the 80 ns SMD simulation of FXIIIB dissociating from FXIIIA for the all atom partial heterotetrameric FXIIIIA_2_B (S1–S4 S8–S10)_2_ model. (Negative correlation, yellow; and positive correlation, blue). The DCCM map is shown only for the FXIIIA residues. Mean correlation (DCCM) values for FXIIIA residues on the FXIIIIA: FXIIIB interface comprising the three calcium binding sites are tabulated on the right for two regions; residues 1–171 (N-terminal beta sandwich domain) and residues 172–692 (the remaining C-terminal domains). (Panel **B**) Separation plots showing physical interactions (blue spots represent interaction at each simulation snapshot; same shade represent interactions at the same snapshot) for residues (x-axis) of one FXIIIA monomer at the FXIIIB (S1–S4 antiparallel S8–S9) interface over the progress time (y-axis) of the SMD (residue numbering for both plots according to the complete zymogen primary sequence). (Panel **C**) Change in mean estimated solvation energies for FXIIIA residues at the FXIIIA: FXIIIB heterodimeric interface during the 80 ns SMD simulation run. The error bars represent standard deviation.

Simulated dissociation of the modeled partial monomeric component of the FXIIIB subunit from the monomeric FXIIIA structure using our SMD method resulted in a sharp change in estimated solvation energy for residues which spatially surround the three calcium binding sites (Fig. 6, Panel C). From a calculated dynamic cross correlation matrix (DCCM) for the simulation we infer that the three calcium binding site residues show a negative correlation with beta sandwich domain residues of FXIIIA subunit interacting with the FXIIIB subunit (Fig. 6; Panel A). In contrast, the residues from the other C-terminal domains (Barrel domains) of the FXIIIA subunit that interact with the FXIIIB subunit show a strong positive correlation. This indicates that the motions influenced by calcium binding at the N- and C-terminals are opposite to each other, suggesting that the entire FXIIIA molecule will tend to twist. This twisting movement of the FXIIIA subunit suggests a mode for weakening FXIIIA: FXIIIB subunit interactions within the heterotetramer. The last FXIIIA subunit residues to lose contacts with the FXIIIB subunit during this simulation are Asn175, Asp297 and Lys677 (Fig. 6, Panel B). Interestingly, simulated dissociation of the FXIIIB subunit from the FXIIIA subunit also distorts the region around the FXIII-AP cleavage site lowering the accessibility around the Arg37-Gly38 sessile bond (Fig. 5, Panel D).

### Presence of the FXIIIB subunits accelerates the rate of *in vitro* activation of FXIIIA_2_

Results of the *in vitro* FXIIIA activation assay (see Supplementary Methods)^52^ revealed that, in the absence of FXIIIB, the rate of generation of FXIIIAa is lower (ka = 0.145, where ka is constant which describes the absorption of FXIIIa development in a one compartment model) than when FXIIIB is present (ka = 0.485). The lag time in FXIIIAa generation assay was shortened by 9.45 minutes when rFXIIIB (Zedira, Darmstadt, Germany) was added to rFXIIIA in FXIII-deficient plasma (Figure S9). The time course for FXIIIAa generation for rFXIIIA in the presence of added rFXIIIB subunit resembled that of normal pooled plasma.

### The FXIIIB subunits separate from the FXIII heterotetramer at high calcium ion concentration in the *in vitro* activation assay

**Figure 7:**
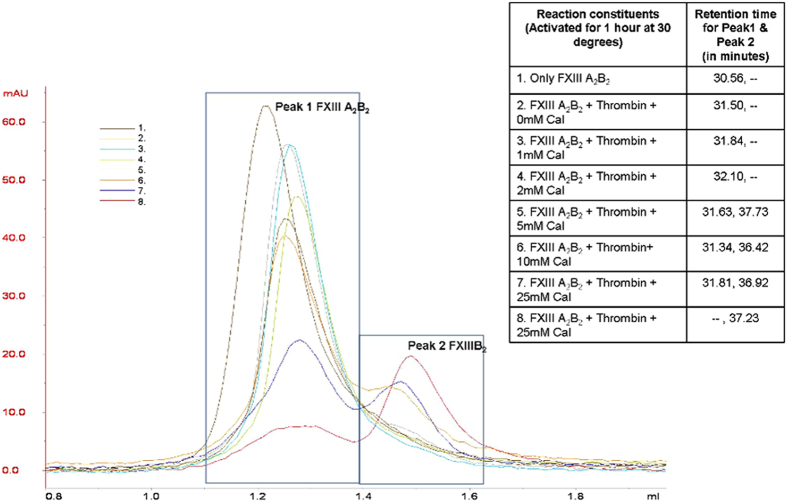
*In vitro* dissociation of the FXIIIA_2_B_2_ heterotetramer observed by gel filtration. Gel filtration elution profiles for purified heterotetramer FXIIIA_2_B_2,_ at various calcium & thrombin concentrations in local environment during *in-vitro* activation. The color key (with number) depicts the different treatment conditions during activation (number corresponds to the table on right). X-axis shows the retention volume of peak; y-axis shows the amount of protein in mAU. The table also details the retention time of the peaks corresponding to FXIIIA_2_B_2 (peak 1)_ and FXIIIB_2 (peak 2),_ at different conditions, respectively. Protein identities eluted at peak maxima confirmed by mass spectrometry (Supplementary Figure S10).

Gel filtration analysis (Fig. 7) revealed that only at very high calcium ion concentration (25 mM) during activation, measured *in vitro* concentrations of FXIIIB subunit suggest that it completely dissociates from the initial FXIIIA_2_B_2_ complex (peak separation starts at *in vitro* calcium ion concentration: 5 mM/activation reaction mixture of 50 μl). The first eluted peak corresponded to the tetrameric FXIIIA_2_B_2_ complex as determined by mass spectrometry, while the second peak contained only FXIIIB_2_ (Figure S10). No dissociated FXIIIA_2_ or activated FXIIIAa was observed in any of the eluated peaks. The second peak corresponding to FXIIIB appeared distinctively when the concentration of calcium ions exceeded 5 mM in the reaction mix applied to the column. In the absence of thrombin cleavage, however, no separation of subunits was observed irrespective of calcium ion concentration (0–25 mM). The resolved FXIIIA_2_B_2_ and FXIIIB_2_ peaks at different calcium concentrations exhibited different elution times (Fig. 7).

## Discussion

The activation of FXIII *in vivo* follows a sequence of events that is initiated by proteolytic cleavage, calcium binding and ensuing conformational changes. In the present report we attempt to rationalize the complete train of molecular events with an eye on plausible sub-molecular conformational changes that may take place during this process. The structural and sequence alignment of FXIIIA subunit to the better characterized homologous TG2 transglutaminase suggests a few insightful observations. Firstly, both enzymes share identical domain organization except for an additional activation peptide (FXIII-AP) absent in TG2 and which is cleaved by thrombin in the first step of *in vivo* FXIII activation. *In vivo*, TG2 remains inactive until binding of GTP/GDP at low intracellular calcium levels^55^. At relatively higher extracellular calcium concentration and low concentration of GTP/GDP, a redox switch involving two vicinal cysteine’s (Cys408-Cys409) regulates TG2 activation^56^. The homologous region of FXIIIA, where the vicinal cysteine’s are substituted by Arg408-Cys409 of FXIIIA, forms the central core of the dimeric interface of FXIIIA_2_ and is also part of the buried Cab3 site in the zymogenic FXIIIA subunit structure. Therefore, this homologous region of FXIIIA appears to have evolved as part of a regulatory mechanism (instead of a redox switch) that stabilizes the dimeric interface, occluding the solvent access to the Cab3 binding site. Residues involved in GTP/GDP binding of TG2 (Arg478/Ser482) align with a secondary thrombin cleavage site (Lys513/Ser514) of FXIIIA which suggests that this region interacts with the FXIIIB subunit, instead of GTP/GDP, to prevent secondary cleavage of zymogenic FXIIIA (Figure S1). Therefore, these two homologous regions apparently have regulatory functions in protecting/maintaining the zymogenic form of either protein, although the biochemical nature of the binding partner is different for the two transglutaminases.

Our results suggest that the calcium binding sites of TG2 and FXIIIA serve to coordinate and stabilize conformational changes during a multistep activation process that directs a global change in the respective protein folds and leads to exposure of enzymatically competent active sites. We observed a mean positive dynamic correlation between the movement of different calcium binding site residues with respect to each other when simulating activation of the zymogenic TG2 and the FXIIIA structures. This suggests mutual interdependence and possibly cooperativity between these calcium binding sites during the activation of both structures (Figure S2). Interestingly though, unlike TG2 in which all individual cross-correlations between individual calcium binding sites were positive, in FXIIIA the individual cross-correlation of Cab1 with Cab2 and Cab3 [C (*i*, *j*) = −0.127 and −0.199] each were negative values which was compensated by a high positive correlation [C (*i*, *j*) = 0.886] between Cab2 and Cab3 value resulting in a positive mean correlation value [C (*i*, *j*) = 0.186] (Figure S2). Therefore unlike TG2, the saturation of calcium binding sites for FXIIIA may be a sequentially ordered set of events which is also consistent with conclusions drawn from our transition state intermediate model analysis (Fig. 5). In fact, the constitutively calcium-occupied Cab1 site of FXIIIA may impart a stabilizing influence on the zymogen structure, similar to the effect of GTP/GDP binding on TG2 intracellularly^55^. We observed that when we simulated dissociation of this calcium, there appeared to be an allosteric effect on the region around the FXIII-AP that suggests it may change conformation to a random coil from an ordered beta sheet. The same distortion was also observed in a plain simulation of the FXIIIA subunit 1 ggu file deprived of its bound calcium. On the other hand a plain simulation of the original calcium bound structure did not show the breakdown of the ordered secondary structure clearly indicating that the reason for the loss of order in the region around the activation peptide (even when the rest of the structure does not change much) is the absence of calcium at Cab1. Such a loss of secondary structure would tend to render it more susceptible to proteolytic attack (Fig. 1). This data explains an earlier observation in the literature, which reports that mutating the Cab1 binding site residues results in faster proteolysis of the zymogenic FXIIIA^57^.

Post thrombin cleavage, the Cab1-mediated zymogenic constraint is apparently lost when the Cab2 binding site becomes exposed and coordinates a calcium ion. The first four of a simulated temporal series of eight transition state intermediate models suggest that the co-ordination of the first two calcium binding sites may be antagonistic to each other (*i.e.*, the bound calcium occupancy at Cab2 transiently destabilizes the Cab1 site) (Fig. 3). The cleavage of FXIII-AP may serve multiple roles. Firstly, it may expose Cab2 as discussed above. Secondly, SMD simulated dissociation of the subunits of the zymogenic FXIIIA_2_ dimer (with or without N-terminal FXIII-AP sequence) suggested that the absence of FXIII-AP results in a weakened dimeric interface (Fig. 2). A similar, supportive experimental observation was recently reported by Schroeder *et al.*^32^ using mutated FXIII-AP variants^58^. Lastly, the removal of FXIII-AP, according to our simulation analysis, may result in overall tendency towards heterotetramer dissociation, as supported by analysis of our partial heterotetramer model (Fig. 5). Our simulation analysis also suggests that the ionic co-ordination of Cab2 and Cab3 imparts a conformational change through forces on the peptide backbone that propagates N- to C-terminal, finally enabling the barrel-1 and barrel-2 domains to rotate/translate and convert the apoenzyme to the active form. The calcium coordination of the Cab2 site also confromationally alters residue conformations at the Cab3 site. Stieler *et al.* (2013) hypothesized that this rearrangement of the loop is critical for access of the K substrate (Lysine substrate) that enters the catalytic hydrophobic pocket from this direction^26^. We observed in our partial heterotetramer model that this region is masked partly by portions of the FXIIIB subunit. The rearrangements in the Cab3 site would potentially also weaken the FXIIIA: FXIIIB interactions, thereby increasing access of calcium in the aqueous mileu to the Cab3 site. With our transition state intermediate models we were able to reproduce and observe the calcium binding changes postulated by Stieler *et al.* (2013), but in an all-atoms dynamic representation across a short temporal window^26^. Furthermore, we were able to observe the formation of a hydrophobic catalytic pocket in which the acylenzyme Q substrate (Glutamine substrate) intermediate could be formed. The formation of a hydrophobic tunnel and the oxyanion hole that retains and stabilizes this intermediate was also observed in later stage transition intermediates (Fig. 4B). Such oxyanion holes have been reported not only for FXIII, but also in other transglutaminases as well as cysteine proteases that comprise a family of enzymes from which transglutaminases are thought to have evolved^59^. Movement of the His342-Glu401 diad residues towards each other in the early stage simulation intermediates, and then away from each other in the final stages, was also observed in this study (Fig. 4A). Maurer *et al.* (2004 and 2007) observed that FXIIIA residues 513–522 showed decreased surface accessibility post-activation for both proteolytic and non-proteolytic activation pathways^60^,^61^. Among our simulated transition state intermediates, we observed that residue patch 501–522 in the zymogenic FXIIIA forms a V with an angle of 122° that closes upon itself in subsequent activation intermediates, thereby occluding access to residues 513–522. This would explain the decreased surface accessibility. Similarly, Maurer *et al.* (2004 and 2007) also showed that residues 523–546 also exhibited decreased accessibility only during non-proteolytic activation^60^,^61^. We believe that during non-proteolytic activation, the presence of FXIII-AP serves to retain the activated form of FXIIIA as a weak dimer, unlike in proteolytic activation where it apparently dissociates into monomers. We observed that, on application of simulated forces to separate the FXIIIA subunits, the dimer remains associated only through a few residues (Arg11, Arg12, Asp343 and Glu410) that form interactions between the FXIII-AP and the core domain. Our dissociation simulation results suggest that these associations will have the effect of rotating each monomer relative to the other during activation. As a result, residues 526–546 of one monomer would end up opposed to the analogous residues on the opposite monomer, effectively forming a rearranged interface for the weakened dimer and concomitantly preventing local solvent accessibility (Figure S11). This is also consistent with non-proteolytic activation of FXIIIA being reversible^62^.

When we simulated dissociation of the FXIIIB subunit from the FXIIIA subunit in the partially modeled heterodimer, we observed that structural rearrangements in the interface regions between FXIIIA and FXIIIB showed a strong correlation with rearrangements in the three calcium binding sites along with large variations in estimated solvation energies (Fig. 6). These two relationships in distant structural rearrangements suggest the interdependence of the rearrangements (*i.e.*, the binding of calcium at Cab2 also contributes to weakening of the FXIIIA: FXIIIB interface and also causes local rearrangements at the Cab3 site). The final residues to dissociate in the FXIIIA dimer are from the core domain (Asn 175, Asp297) and barrel-2 domain, (Lys677) indicating that dissociation of at least a portion of the FXIIIB subunit is crucial to full exposure of the catalytic site as well as to the final movements of the barrel domains.

Our simulation suggests that saturation of calcium binding at Cab3 is likely the last molecular event leading to dissociation at the FXIIIA: FXIIIB interface. This is in agreement with our *in vitro* dissociation results observed by gel filtration when higher calcium concentrations resulted in increased dissociation of FXIIIB_2_ dimer from the primary heterotetramer FXIIIA_2_B_2_ (Fig. 7). The absence of FXIIIAa, as a resolved peak in our gel filtration results could be the result of subsequent inactivation or instability of FXIIIAa. This instability of FXIIIAa may be due to dissociation of FXIIIB subunits that would expose the secondary thrombin cleavage site of FXIIIAa 515–516^63^. Interestingly, when we analyzed the exposed surface area of activated FXIIIAa compared to that of the zymogenic form in our simulations, we identified a number of putative sites within additional surface exposed locations that would theoretically be susceptible to protease activity (Figure S12). The FXIIIA zymogenic form, as well as the activated form, has been shown in the literature to be susceptible to cleavage by proteases other than thrombin^2^,^64^. Also, cleavage by polymorphonuclear granulocyte proteases (PMN) has been suggested to be one of a number of possible down-regulation mechanisms for inactivating FXIIIAa within the fibrin clot^65^. We expect that activated FXIIIAa in the absence of bound FXIIIB is down-regulated *in vivo* primarily by sequential proteolytic degradation that might be initiated by thrombin. This also explains reports of its short circulating activity half-life in literature and in results of this study as well^66^. This aspect of down-regulation of FXIIIA requires further investigation, however, as we could not identify evidence of proteolyzed FXIIIA following the two major protein elution peaks observed in the present gel filtration experiments. Interestingly a very recent article suggests that Thrombin cleavage could be a means of FXIIIAa downregulation but only after primary cleavage by Plasmin^67^. The fact that our simulation results suggested that the FXIIIB subunit dissociates from FXIIIA only after the calcium binding site Cab3 becomes saturated is experimentally supported by the results of our gel filtration experiments. Specifically, the amount of FXIII heterotetramer resolved significantly decreased with increasing calcium concentration, but also appeared at different retention times which suggest that the complex structure undergoes some conformational transformation as a loose/weakened heterotetramer without the dissociation of FXIIIB subunit.

A secondary observation in this study, which has also recently been reported by two other groups^35^,^36^, was the influence of FXIIIB subunit on the activation of FXIIIA. We observed in FXIII generation assays (which mimic the physiological conditions) that addition of rFXIIIB_2_ to rFXIIIA_2_ accelerates activation (Figure S5). Ichinose *et al.*^31^ explained their similar observation by the interaction of FXIIIB_2_ subunit with fibrinogen^35^. However, in the same article they also elaborate that differences in the FXIIIB_2_ C-terminus (interaction site for Fibrinogen) as observed between FXIIIB subunit isoforms FXIIIB*1 and FXIIIB*3 do not affect fibrinogen binding and that there might be other contributing factors to this observation. We suggest an explanation for this observation using simulation result based on our partial heterodimeric model of FXIIIA_2_B. Specifically, our results suggest that Sushi domain 1 may dock onto a hydrophobic fold formed by the N-terminal part of the activation peptide (Fig. 5). Also, our simulation results suggest that dissociation of FXIIIB may cause the FXIII-AP sessile bond to be less accessible. We suggest that the putative hydrophobic interactions between the FXIIIB Sushi S1 domain and the FXIII-AP may allow increased efficiency in proximal thrombin cleavage. This is evident also from our FXIIIB subunit spiked FXIII generation assay, results where in the presence of FXIIIB accelerates the activation of FXIIIA. This combined with the data from activation peptide, suggests that the interactions of activation peptide with the opposite monomer of FXIIIA and the FXIIIB subunit maintain the region around the activation peptide in a spatial location ideal for insertion and cleavage by thrombin at the right time and place i.e. they both contribute to a perfect cleavage (neither too early nor delayed).

The entire simulated sequence of activation events described in our article would be expected to take place in the plasma (Concentration of calcium: Free ions 1.18 mmol/L, protein bound 1.14 mmol/liter, total 2.48 mmol/liter)^68^. The question then arises: What is the sequence of events involving activation for intracellular FXIIIA_2_? We assume that the dimeric FXIIIA_2_ molecule exists intracellularly as an ion regulated equilibrium between its activated and zymogenic state. Since the intracellular levels of calcium are lower than in plasma, the occluding effects of FXIII-AP and FXIIIB binding on the Cab2 and Cab3 calcium binding sites would not be expected to be consequential *in vivo* in cells. At low levels of calcium, only the Cab1 site would be occupied and, hence, a dynamic equilibrium shift towards the zymogenic state is expected. A change in this equilibrium might be caused by intracellular calcium influx under conditions when activation of intracellular FXIIIA_2_ is required. Therefore, saturation of Cab2 and Cab3 with calcium could occur without FXIII-AP cleavage, resulting in a reversible form of activation of FXIIIA_2_ possibly in a manner similar to our simulated results. Interestingly, it has been reported that FXIIIA_2_ can also be activated without thrombin cleavage and at low calcium concentration, but only on a very long time scale^69^. Thus, our results further suggest that all three calcium binding sites are occupied during the activation of FXIIIA irrespective of the type of activation.

To briefly summarize our work, this study provides some major insights into the activation and regulation mechanism of FXIII. In a stepwise manner we have shown how calcium binding regulates both the zymogenic and activated forms of FXIIIA. The information contained in the subtle structural changes observed in the various intermediates models can be used to design high efficiency inhibitors against FXIII. Additionally, for the first time our study lends a hypothetical structure functional perspective/account of FXIIIA: FXIIIB interactions during the dissociation of the FXIIIIA_2_B_2_ complex. Information in this regard would further contribute to the final elucidation of the FXIIIIA_2_B_2_ complex structure and its dynamics during assembly or dissociation.

## Additional Information

**How to cite this article**: Gupta, S. *et al.* Revisiting the mechanism of coagulation factor XIII activation and regulation from a structure/functional perspective. *Sci. Rep.* **6**, 30105; doi: 10.1038/srep30105 (2016).

## Supplementary Material

Supplementary Information
