# Supplementary material for: Revisiting the mechanism of coagulation factor XIII activation and regulation from a structure/functional perspective
Source: Sci Rep. 2016 Jul 25;6:30105. doi: 10.1038/srep30105 (PMC4958977; doi:10.1038/srep30105)
Supplement: Supplementary Information [file srep30105-s1.doc]

Research Article

**Supplementary file**

**Revisiting the mechanism of coagulation Factor XIII activation and regulation from a structure functional perspective**

Sneha Gupta^†^, Arijit Biswas^1†*^, Mohammad Suhail Akhter^1^, Christoph Krettler^2^, Christoph Reinhart^2^, Johannes Dodt^3^, Andreas Reuter^3^, Helen Philippou^4^, Vytautas Ivaskevicius^1^, Johannes Oldenburg^1*^

^1^Institute of Experimental Haematology and Transfusion Medicine, University Clinic Bonn, 53127 Bonn, Germany

^2^Max planck institute of Biophysics, Frankfurt, Germany.

^3^Paul Ehrlich Institute, 63225 Langen, Germany

^4^Leeds Institute for Cardiovascular and Metabolic Medicine, University of Leeds, Leeds, UK

**Running head title:** Coagulation Factor XIII activation/regulation

^†^These authors contributed equally to this work.

***Correspondence should be addressed to:**

Dr. rer. nat. Arijit Biswas, Institute of Experimental Haematology and Transfusion Medicine,

University Clinic Bonn, Sigmund Freud Str. 25, 53127 Bonn, Germany.

Email: arijit.biswas@ukb.uni-bonn.de

Phone: +49 228 287 19428

Fax: +49 228 287 14320

or

Prof. Dr. med. Johannes Oldenburg, Institute of Experimental Haematology and Transfusion Medicine,

University Clinic Bonn, Sigmund Freud Str. 25, 53105 Bonn, Germany.

Email: johannes.oldenburg@ukb.uni-bonn.de

Phone: +49 228 287 15175

Fax: +49 228 287 14783

**Supplementary methods**

**rFXIIIa cloning, expression and purification in *Pichia pastoris*.**

*Pichia Pastoris* host strains (PichiaPink™ Strain 3 ade2^-/-^, prb1^-/-^) were purchased from Invitrogen (San Diego, CA). Plasmid amplifications were performed in *E. coli* DH5α cells. FXIIIA cDNA was cloned in-frame with or without a *Pichia* secretory signal (short α-mating Factor) upstream of the strong AOX1 promoter. The vector carries the *Ade* gene to aid selection of transformants in histidine-deficient growth media. The gene of interest was flanked with combination of Tags (10X His, Strep, and FLAG) separated by TEV protease cleavage site.

**Determination of FXIII activity of recombinant FXIII-A_2_**

The FXIII activity of samples 1 and 2 (Recombinant FXIII-A_2_: sample1 = FXIII A 0.8 and sample2 = FXIIIA 0.3, generated in house where, Sample 1 & 2 are same constructs of rFXIIIA, expressed and purified in different batches) were determined with the fluorogenic FXIII assay ( 11). Dilutions of samples were added to FXIII-deficient plasma and FXIII activity determined and normalized to dilutions of human standard plasma calibrated against the WHO 1^st^ International Standard plasma (NIBSC code 02/206). Experimental results were evaluated using CombiStats (EDQM, Council of Europe, Strasbourg, France) statistical software according to the parallel line assay model as described in Chapter 5.3 of the European Pharmacopoeia (5th Edition 01/2005 to 8th Edition 01/2014). Mean activities were from six independently measured dilution series. Sample 1 was assigned an activity of 30 IU/mL; sample 2, an activity of 60 IU/mL.

**Purification of FXIIIA_2_B_2_**

FXIIIA_2_B_2_ heterotetramer was purified from *Fibrogamin P* (CSL Behring). The lyophilized protein was reconstituted in 30 mM Tris, 150 mM NaCl, pH 7.4 (Buffer A). The sample was applied to a Superdex 200 10/300 GL Gel filteration coloumn (GE Healthcare,Germany) connected to an AKTA SMART purifier system equilibrated with Buffer A*.* Purification was carried out under non denaturing and calcium free conditions (FigS6 a). Protein recovered in resolved peak fractions was analyzed by Native PAGE (NuPAGE, Invitrogen, Germany), and commassie-stained (ThermoScientific) bands were analyzed by mass spectrometry (peptide mass fingerprinting after in-gel tryptic digest) (Figs. S6 B, C, D). Protein in fractions from the singleFXIIIA_2_B_2_ heterotetramer peak was mono-dispersed and homogenous and recovered protein was re-purified by repeated gel filtration chromatography. Protein was quantified using BCA protein assay (Thermoscientific, Rockford) and stored at -80 deg C.

**Native PAGE**

Electrophorectic separation of proteins was carried out under non-reducing conditions. The sample was mixed in 1:1 ratio with sample loading dye (without DTT) (NuPAGE, Invitrogen, Germany) and resolved on 4-12% bis-tris gels with MES running buffer at 200 V (Starting and ending current: 110–125, 70–80 mA/gel, respectively). After the run, gel was stained overnight in Commassie stain (Invitrogen), following by destaining with deionized water. Band were cut carefully and analyzed by mass spectrometry (peptide mass fingerprinting).

**Mass spectrometric analysis of proteins from gel filtration peak fractions**

Coomassie-stained protein bands were excised and their identity was confirmed as published elsewhere [R1] with modifications. Peptides were eluted with 25mM NH^4^HCO^3^; 10% acetonitrile (ACN) and digestion stopped by adding 5% formic acid. Peptides were resolved on a nano-ultra performance LC system coupled to a nano-ESI-MS (nano Acquity UPLC nanoESI Synapt-MS, Waters, Milford, US) with a 5 µm symmetry 180 µm x 20 mm C18 pre-column and a 1.7 µm BEH 130 100 µm x 100 mm C18 separation column. A 30 minute gradient (3% ACN to 40% CAN at 500 nL/min) after 3 minutes of trapping (99% water at 5 µL/minute) was applied to separate peptides. The MS was operated in V mode, acquiring MS^E^ data and applying standard parameters. Data analysis was performed using ProteinLynx Global Server version 2.4 (Waters), searching an in-house database consisting of the Uniprot database (May 2011 version, restricted to reviewed entries of eukaryotic organisms; taxon identifier 2759). Proteins hits were accepted at a false positive rate of less than 4%.

**Modeling of the partial heterotetramer complex FXIIIA_2_B_2_**

The B subunit or a monomeric form of it was modeled on a threading server ITASSER that is currently ranked first in modeling competition CASP. Usually the ITASSER provides five models and they give their own score. It is calculated based on the significance of threading template alignments and the convergence parameters of the structure assembly simulations and is known as C-score. It is typically in the range of [-5, 2], where a C-score of higher value signifies a model with a high confidence and vice-versa. Also one can run the ITASSER in default mode or specify certain guiding constraints of our own. In our case we used the structural disulfide known within the B subunit to specify guiding constrains of 2.0 Å distance between The `Sγ´ atoms of the participating cysteine’s in the specific disulfide combination. The template and sequence identity details for this round of automated modeling are as follows:


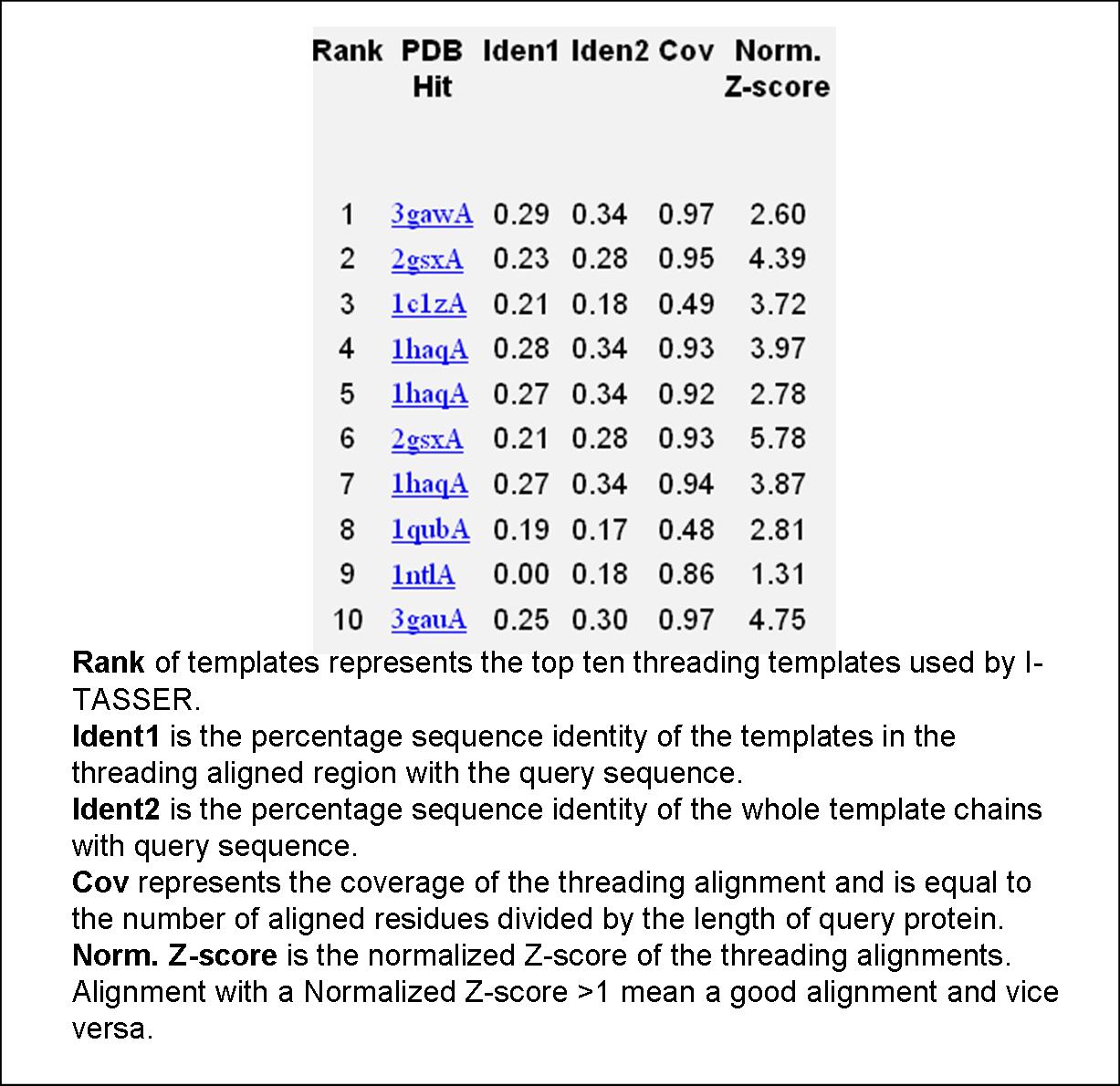


Amongst the models that the ITASSER threw out the best showed a good C-score of 0.56. Based on this score we chose this monomeric model for further analysis. Upon a closure inspection of this model we found that almost all sushi domains were fairly well organized i.e. all sushi domains reported in literature have a highly conserved structural (3+2 beta sheeted structure with variable length loops) core that has only a few variable features. The main problem with this default model was that the N-terminus sushi domain 1 had not been properly been organized into a folded sushi domain and also many disulfide bonds in the respective sushi domains were not in oxidized forms. Also certain beta strands were not in ordered form consistently in all sushi domains. Therefore our next step was to replace these individual sushi domains with high quality models of singular sushi domains that we have previously reported. These models were homology modeled and had been validated based on the template (Complement Factor H sushi domains) as well as for its stereochemical features when we had reported them in extensive details in our earlier publication.(28) However while replacing these models on the sushi domains of the default modeled structure we did not in anyway influence the connecting linker regions to retain some part of the original geometry of the default model. This model was then symmetrically docked. Now for choosing the best dock we used two bits of experimental data from literature: A) Dimerization of the B subunit involves the head to toe arrangement of the filamentous B monomers through the sushi domains 4 and 9 B) Sushi domain interactions are by and large electrostatic in nature. (29, 52) Therefore when we analyzed the docking possibilities we screened for docks which had proximal 4 and 9 sushi domains in a head to toe arrangement. Interestingly if one looks at the surface electrostatic charge distribution on the monomeric models one can observe two distinct symmetrically fitting and oppositely charged regions involving sushi domains 4 and 9(see figure below). Symmetrical docking on M-Z docking server generates 10 best fitting docks (based on the server’s individual clustered scores). Luckily amongst these ten docks only one dock (6th docking pose) showed a fitting involving the regions and in a head to toe manner as suggested above. Therefore this dock was naturally selected as the best dimeric model. Initially we attempted to dock this structure on the zymogenic crystal structure of FXIIIA on the Z docking server. However, owing to the large size this attempt resulted in failure. Therefore we then used intuitive modeling based on what is known about FXIIIA and FXIIIB interaction in order to generate the final complex. The FXIIIB subunit length and total surface area (based on measurements of individual sushi domains) is approximated to be almost same as that might be required to wrap FXIIIA dimeric subunit surface completely (28) and the high association values (28, 30) suggest that the binding is cooperative with the involvement of multiple sushi domains with the A subunit but so far only sushi domains 1 and 2 have been shown to take part in this interaction. With these ideas in order to be able to use the Z docking server we docked a symmetrical half of the FXIIIB_2_ dimer model lacking sushi domains 5,6 and 7 on the symmetrical half of the A subunit zymogenic structure. Now the criteria for selection of the best docking pose was interaction of Sushi domains 1 and 2 or both with the A subunit half (monomer). Only one docking pose out of the best (i.e. high scoring) 10 docking poses showed this orientation and therefore was our final choice for further generating the partial hetero-dimeric model. Assembling the final heterotetrameric form was easy as the core FXIIIA dimeric interaction residues are already known from the dimeric zymogenic FXIIIA crystal structure. Using these residues as guiding constraints we once again symmetrically docked the hetero-dimeric A and partial B combination to generate the final partial heterotetrameric form. When we superimpose the partial heterotetrameric model on the zymogenic FXIIIA dimeric crystal structure it aligns perfectly showing an RMSD of only 0.261Å in the structural alignment for the A subunit region. Each selected model from the several docking poses at each step of modeling as shown in Figure S6 was subjected to rounds of plain and model simulation mentioned individually in the methods section of the main file.(40) The final complex was subjected to a plain MD simulation (100 ns) run in order to test its stability (Figure S8). We also tested the stereochemical quality of the heterotetrameric complex model by submitting it to the MOLPROBITY server (<http://molprobity.biochem.duke.edu/>) usually used for X-ray based structure. The parameter boxes colored green are in excellent agreement while yellow ones are in good agreement for adequate stereochemical features. The results are as below:

**Supplementary Figures**

**Figure S1: Comparison of TG2 and FXIIIA subunit structures and sequences**


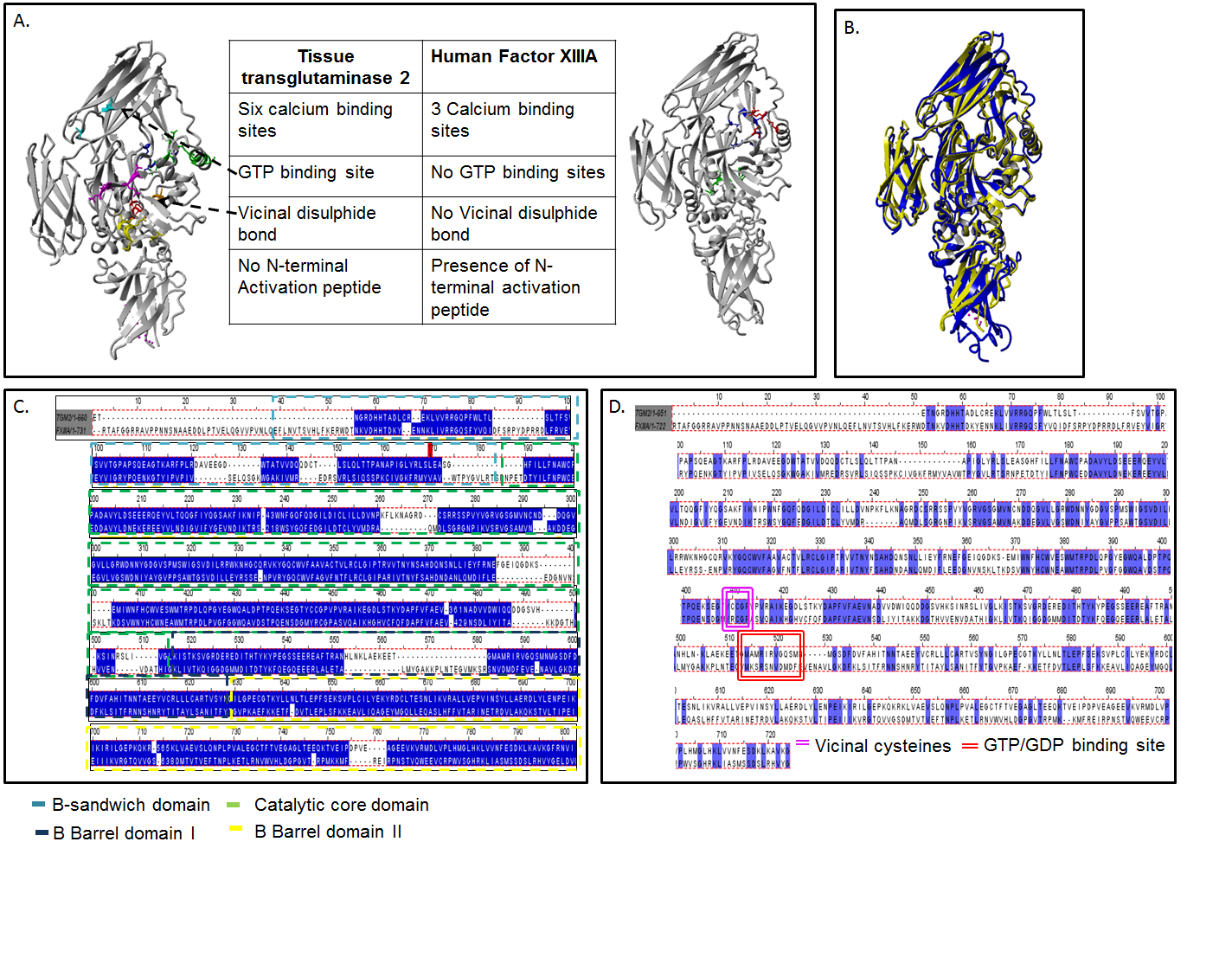


**Panel A:** Tabulated feature differences between FXIIIA (right) and TG2 (left) activation regulators.

**Panel B:** Alpha-carbon backbone trace (ribbons) of the structural alignment of TG2 (yellow) with FXIIIA subunit monomeric chain A(blue).

**Panel C:** Primary sequence alignment corresponding to the structural alignment for TG2 with FXIIIA shown in Panel B.

**Panel D:** A sequence alignment of TG2 and FXIIIA subunit performed using MAFFT with default parameters.

**Figure S2: RMSD variations during plain simulation runs of the PDB file 1f13 (Panel A) and 1kv3 (Panel B) at three different temperatures (298 K, 340 K and 370K).**

**Panel A**


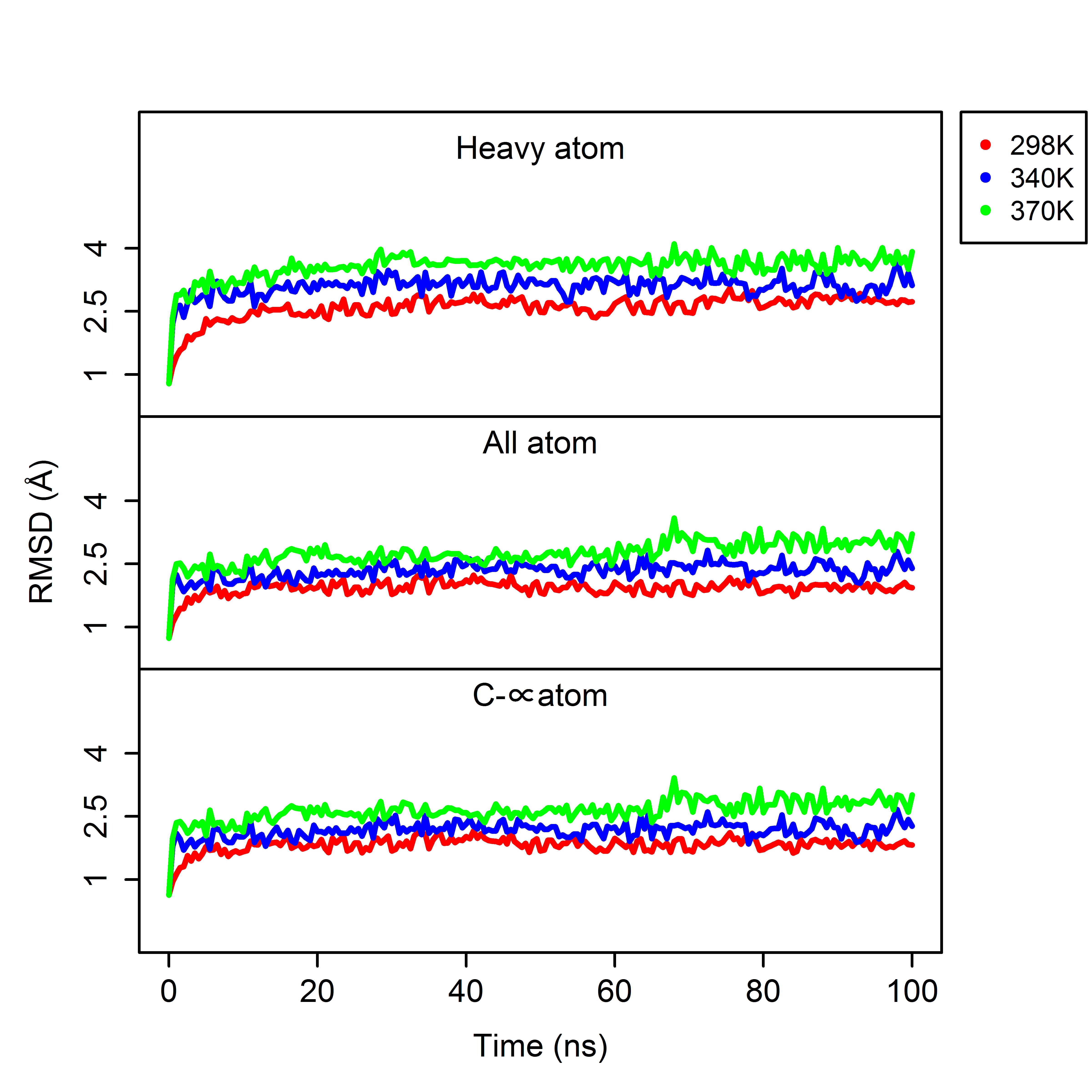


**Panel B**

**
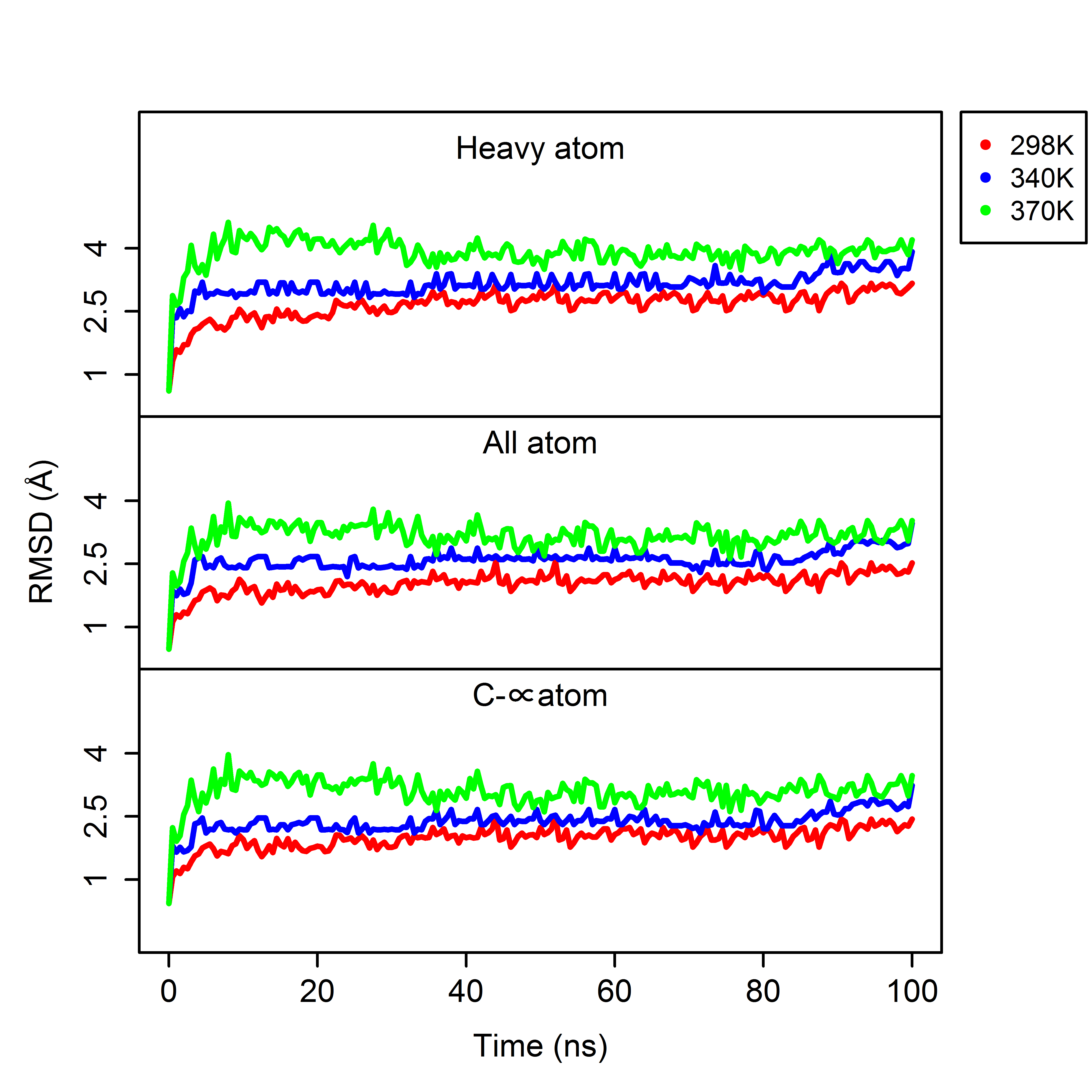
**

**Figure S3: Classical molecular dynamic simulation of FXIIIA_2_ subunit and TG2 structures**

**
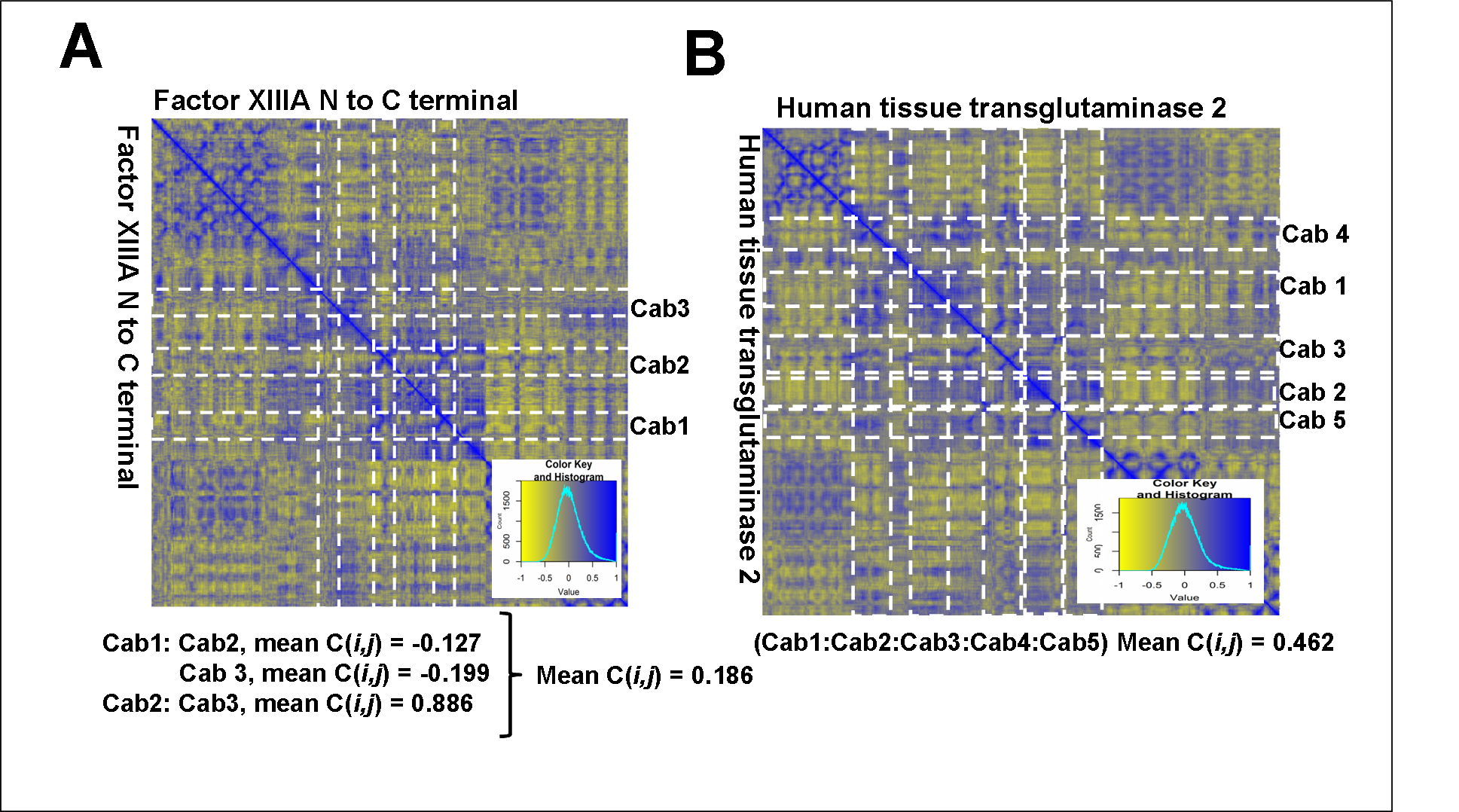
**

**Panel A:** Inter-residue displacement (DCCM) correlation calculated for the 100 ns MD simulation of zymogenic FXIIIA subunit structure (PDB file: 1f13).

**Panel B:** Inter-residue displacement (DCCM) correlation calculated for the 100 ns MD simulation of zymogenic TG2 structure (PDB file: 1kv3). Negative correlation, yellow; positive correlation, blue.

**Figure S4: RMSD variations during plain simulation runs of the PDB file 1ggu (with calcium) (Panel A) and 1ggu without calcium (Panel B) at three different temperatures (298 K, 340 K and 370K).**

**Panel A**


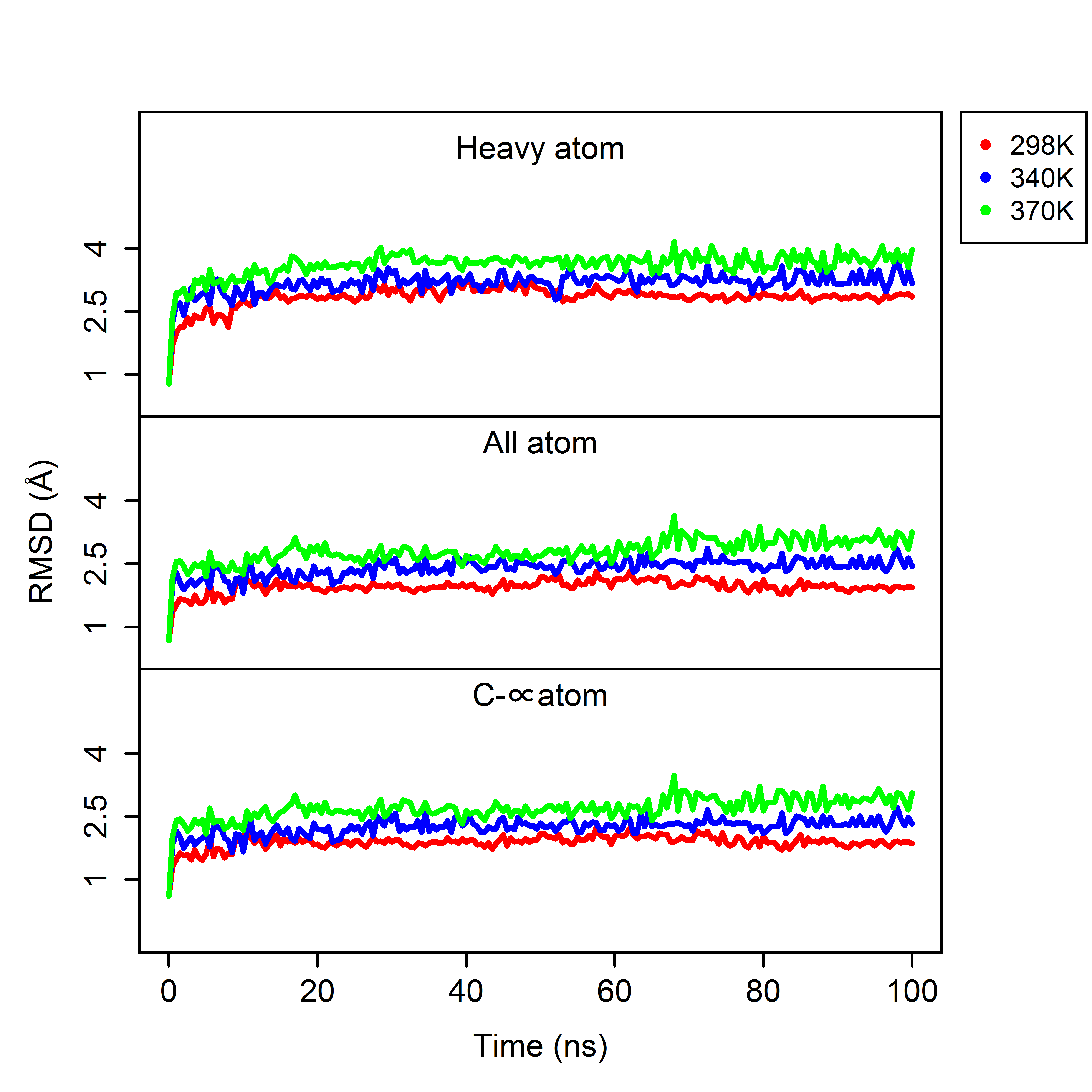


**Panel B**


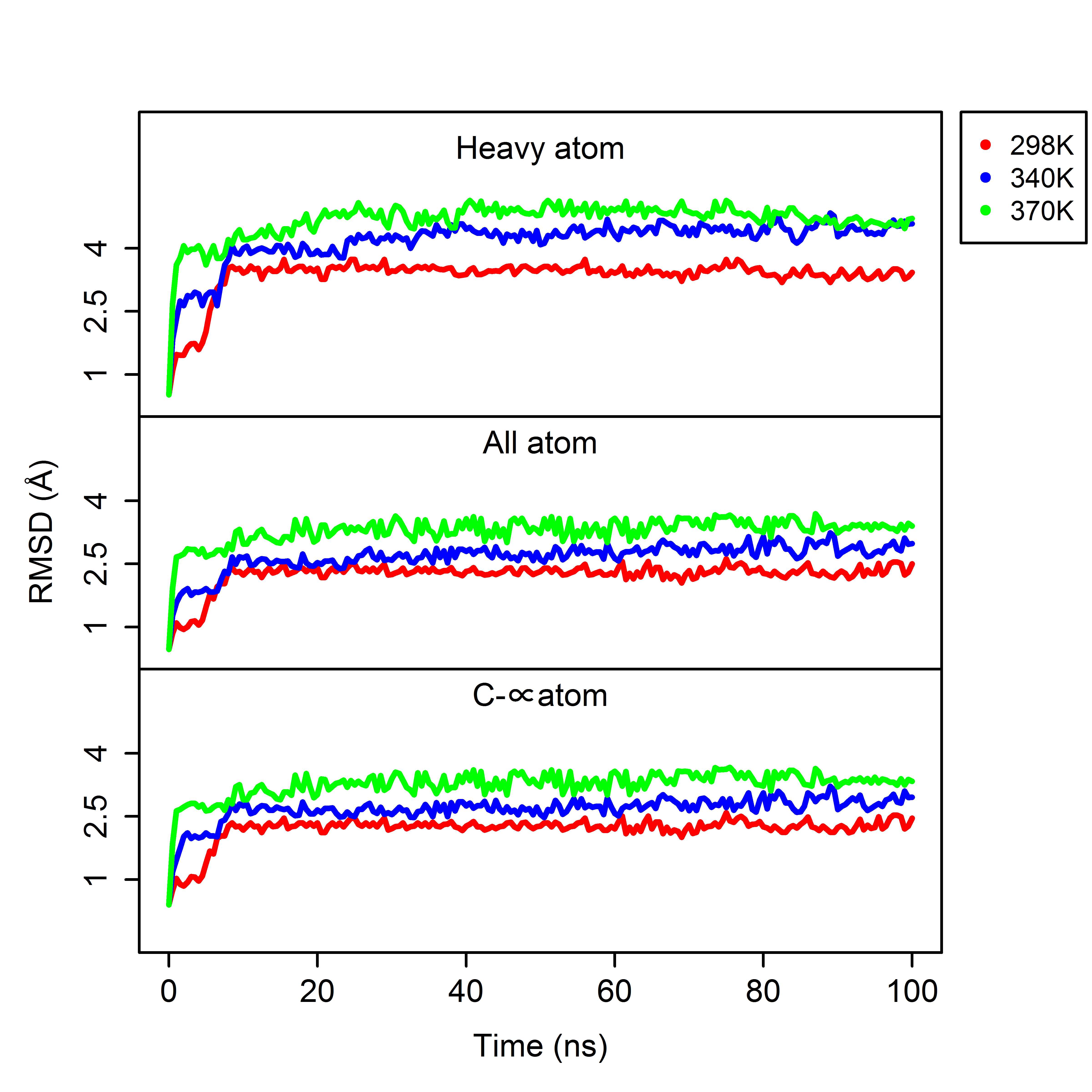


**Figure S5: Secondary structure predictions for the 8 transition state intermediate models**

**
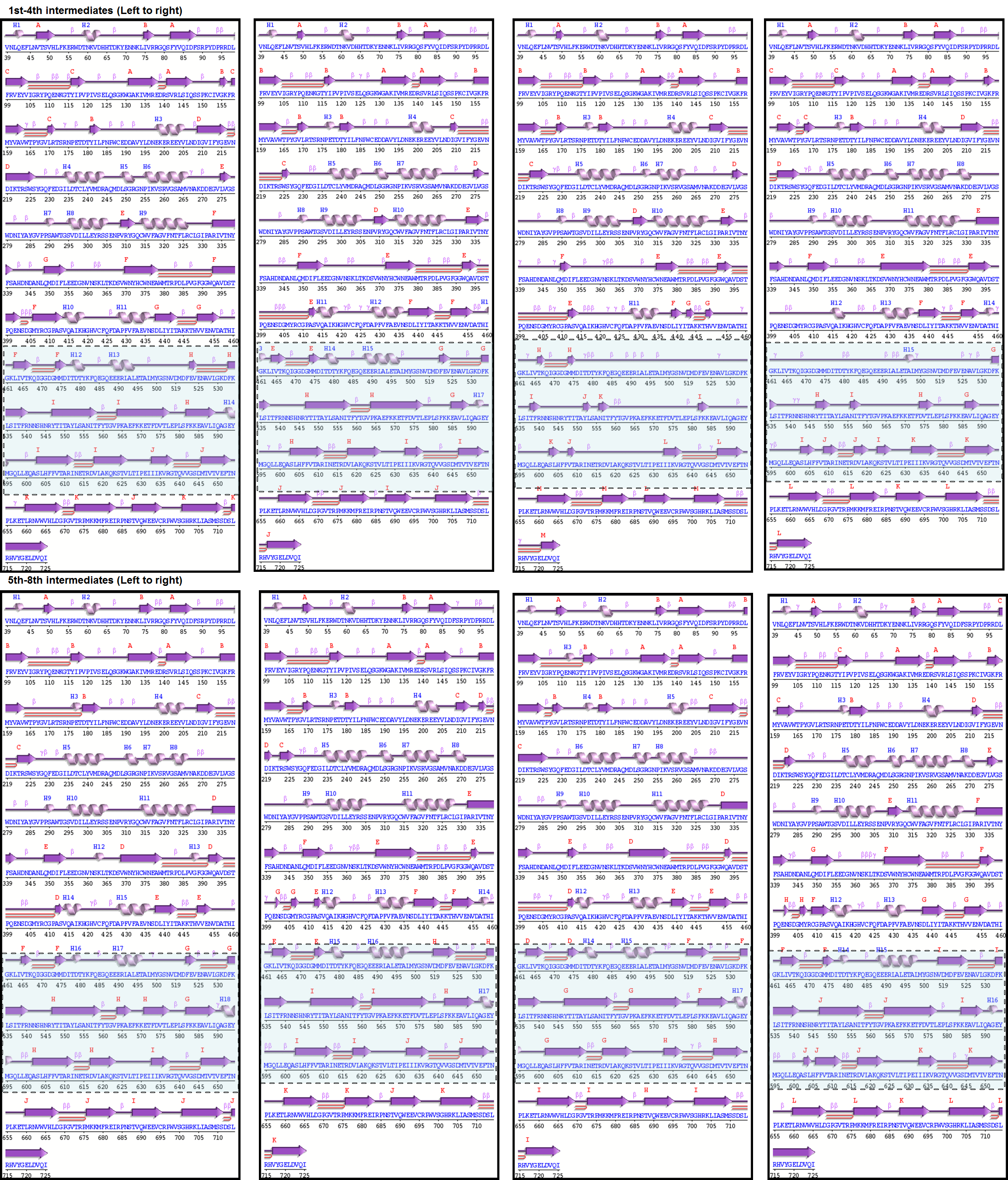
**

This figure illustrates the secondary structure of the intermediate and end state structure models generated for the activation pathway. The figures were generated on the Profunc server (<https://www.ebi.ac.uk/thornton-srv/databases/profunc/> accessed on 05.02.2015). Image descriptions are available here:<https://www.ebi.ac.uk/thornton-srv/databases/profunc/doc/profunc_tutorial.pdf>. The shaded regions represent areas of major secondary structure change.

**Figure S6: Construction of the FXIIIA_2_B_2_ partial heterotetramer model.**

**
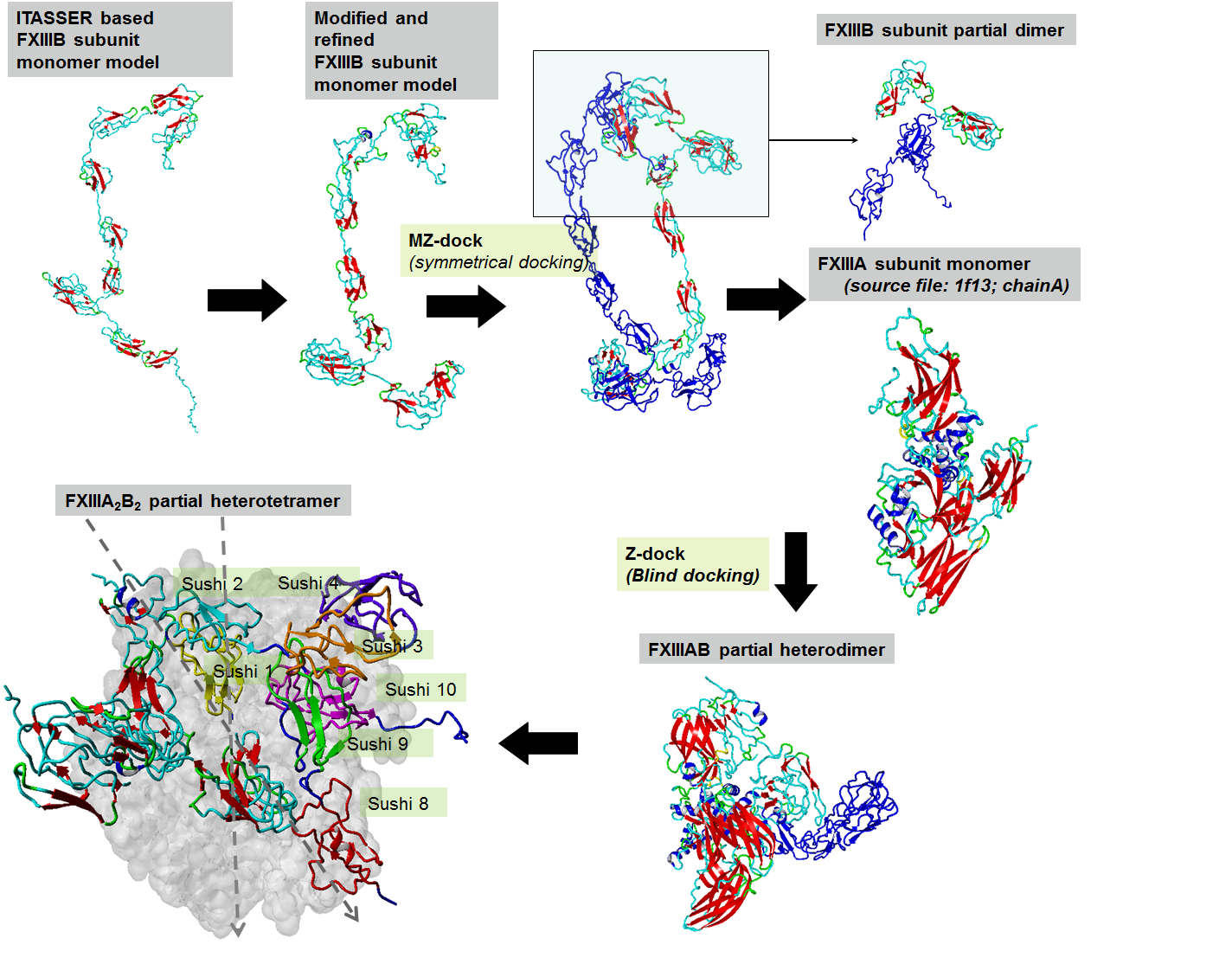
**

This figure illustrates the step wise construction of the FXIIIA_2_B_2_ partial heterotetramer model. The details have been described in the methods section.

**Figure S7: A surface electrostatic description of the FXIIIB monomers showing the two oppositely charged electrostatic patches by which they bind to each other to form a dimer.**


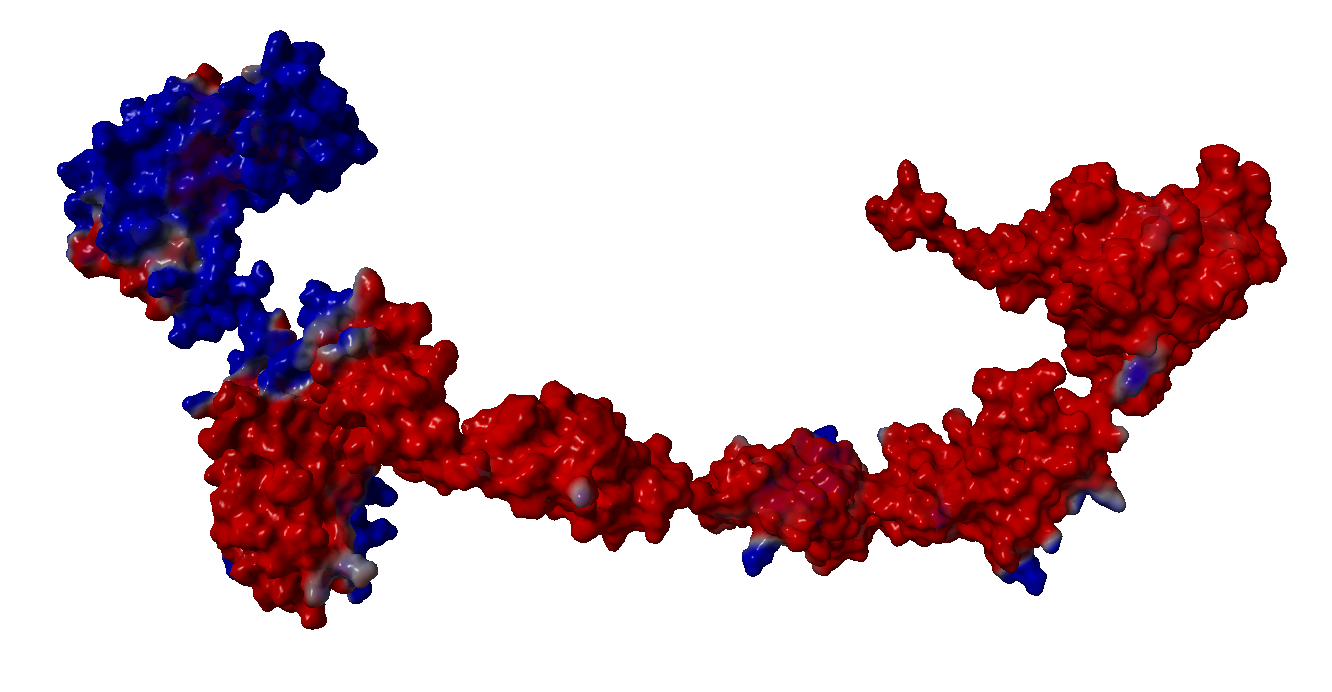

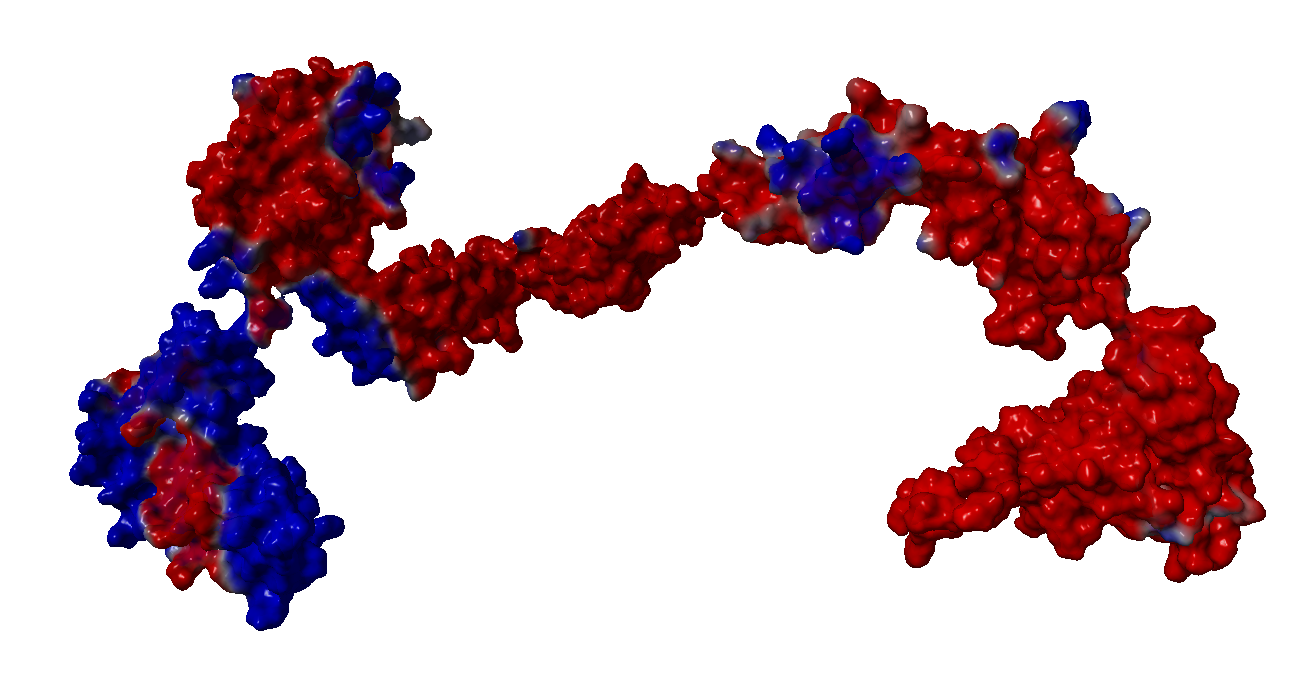


**Figure S8: The RMSD graph for the simulation runs of the partial FXIIIA_2_B heterotetrameric model performed at three different temperatures(298 K, 340 K and 370 K).**


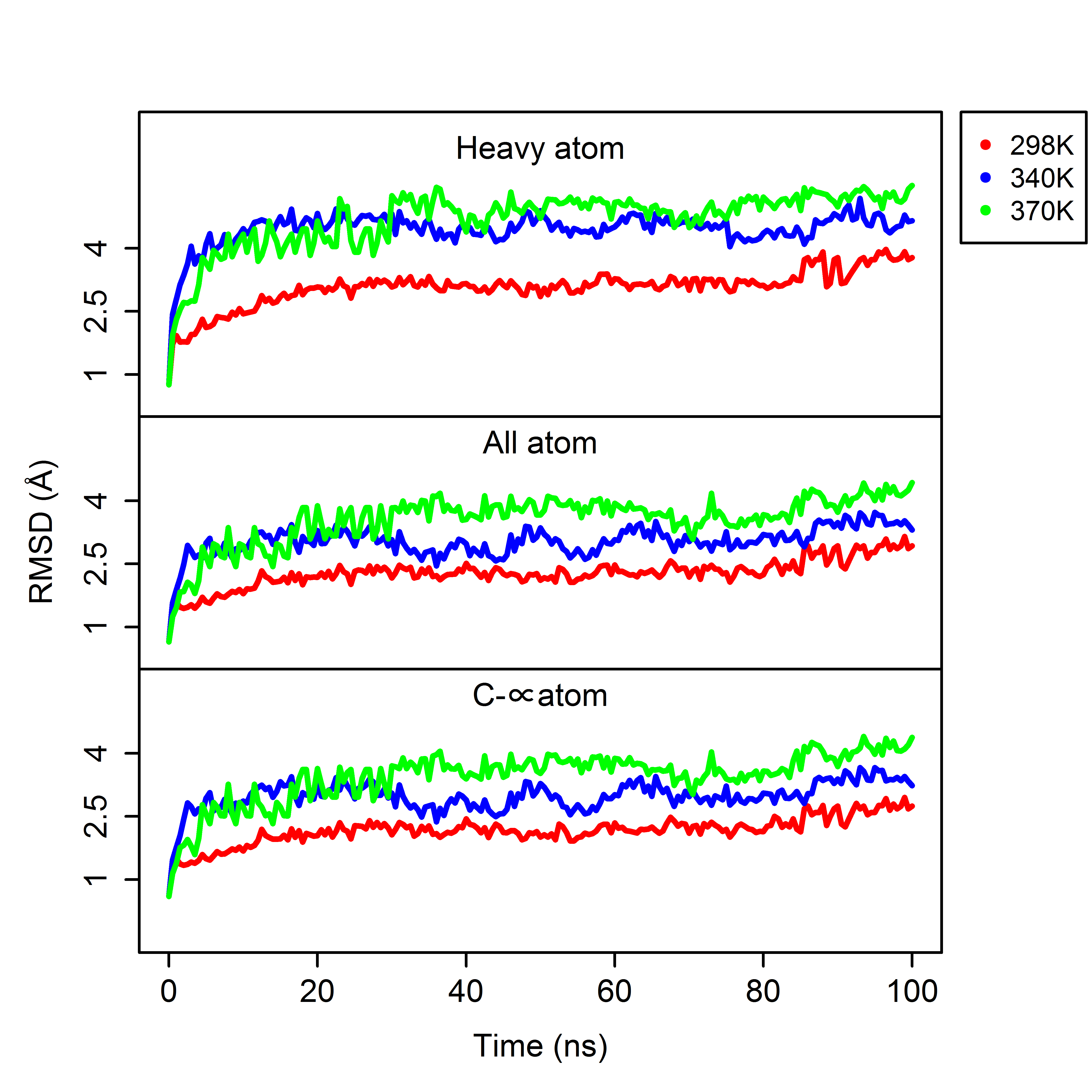


**Figure S9: Effect of increasing concentrations of rFXIIIB_2_ subunit added to the FXIIIAa generation assay**

**
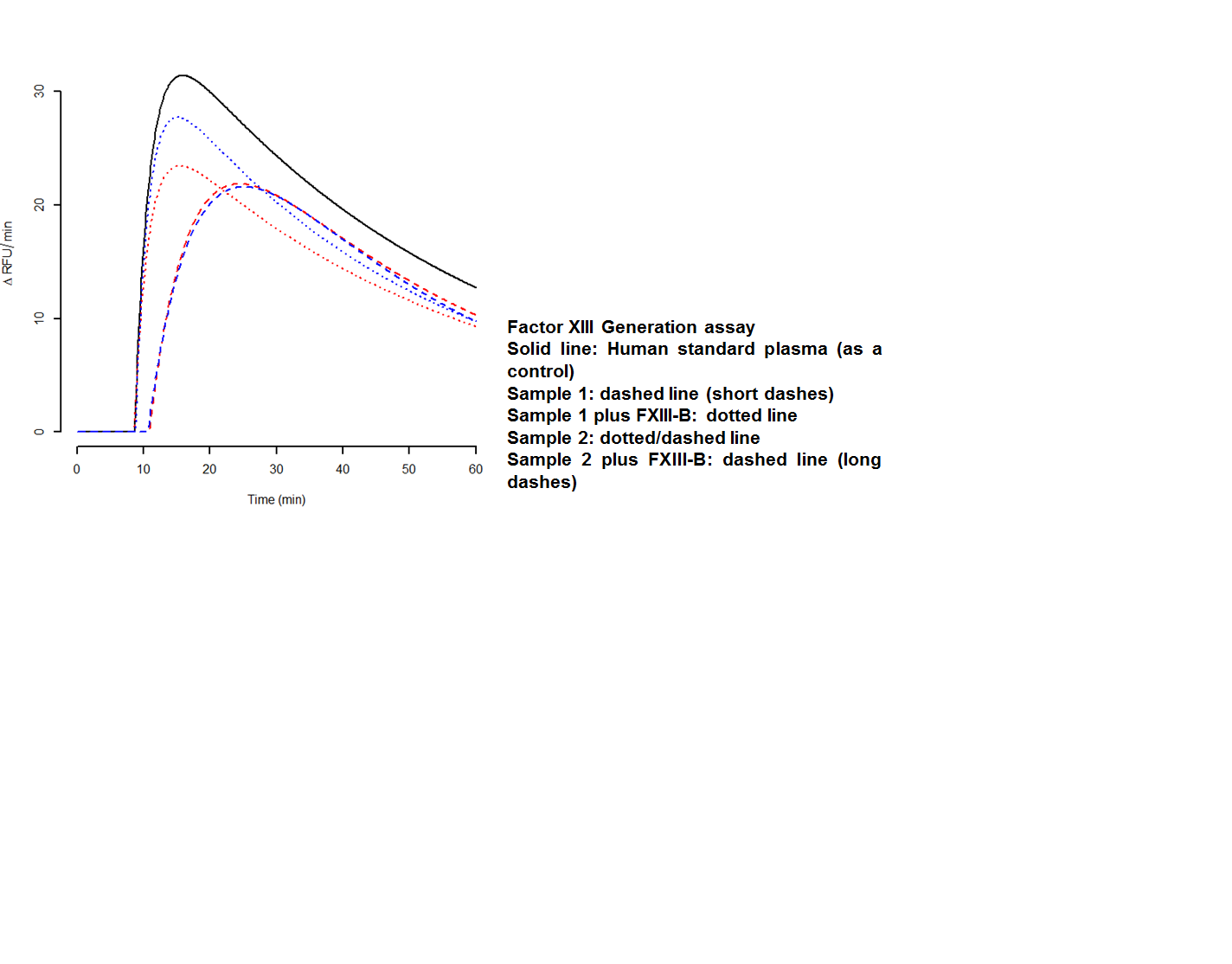
**

This figure explains the effect of presence rFXIIIB, on the activation of rFXIIIA in FXIII generation assay. X-axis denotes the time (min) on activation, and Y-axis denotes the Flouroscence of chromogenic substrate (RFU/min) as the reaction progresses. The rate of generation of active FXIIIAa increases on addition of rFXIIIB in the background of FXIII deficient plasma.

**Figure S10: Mass spectrometry results identifying proteins in peak fractions from gel filtration elution of purified FXIIIA2B2 heterotetramer**

**
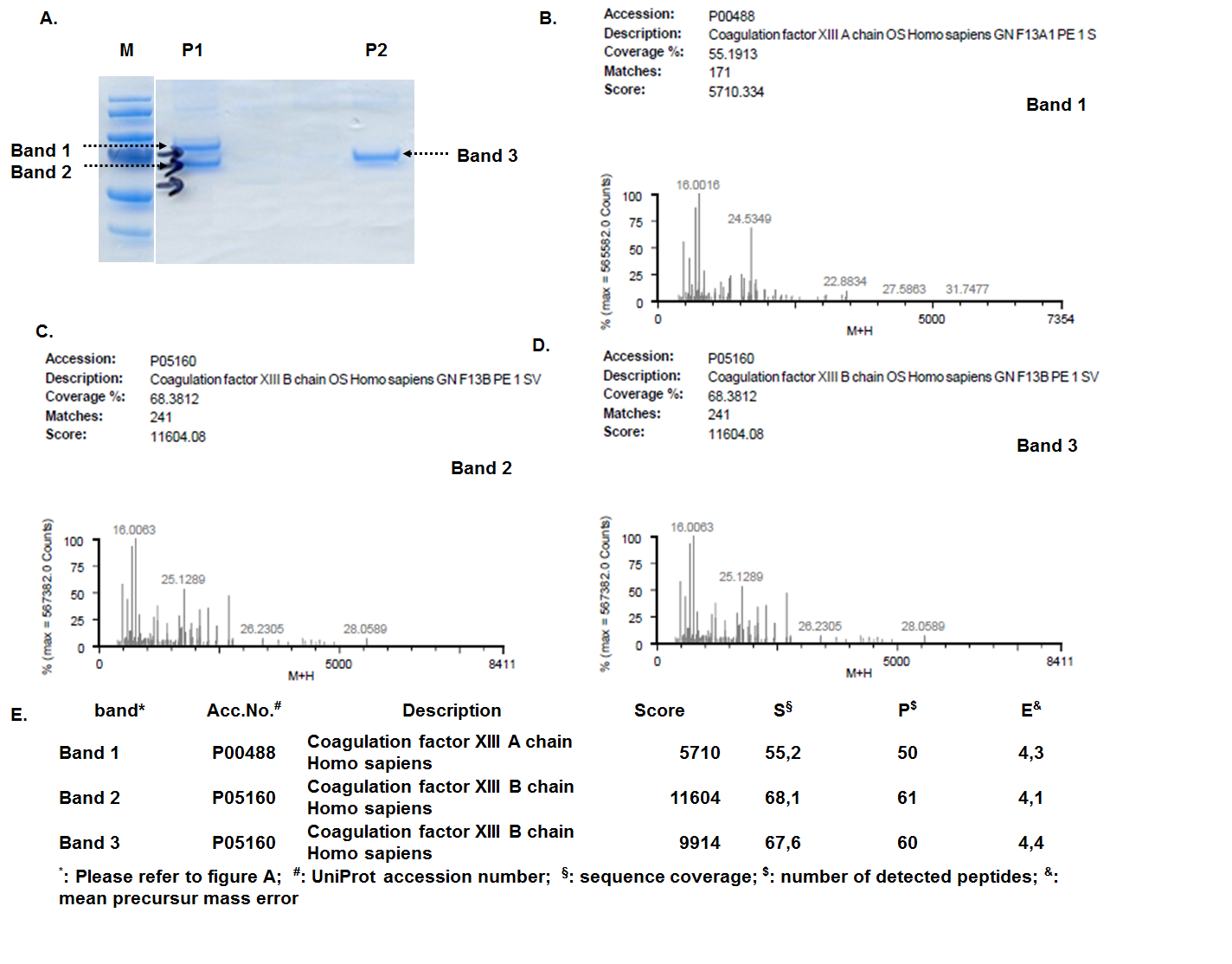
**

**Panel A.** Behavior of peaks separated and eluted from gel filtration(SEC) runs when run on NATIVE PAGE (from FXIII activation assay runs as explained in figure 7), Where P1 is peak 1, and P2 is peak 2. Different resolved bands were marked 1, 2 and 3 as shown in figure. **Panels B, C, and D,** show the mass spectrometric analysis of the individual peaks. **Panel E.** table depicts the description of the protein detected in majority in each of the bands (depending on the score, complete list is not shown here).

**Figure S11: Model of a hypothetical non-proteolytically activated FXIIIA subunit weak dimer**

**
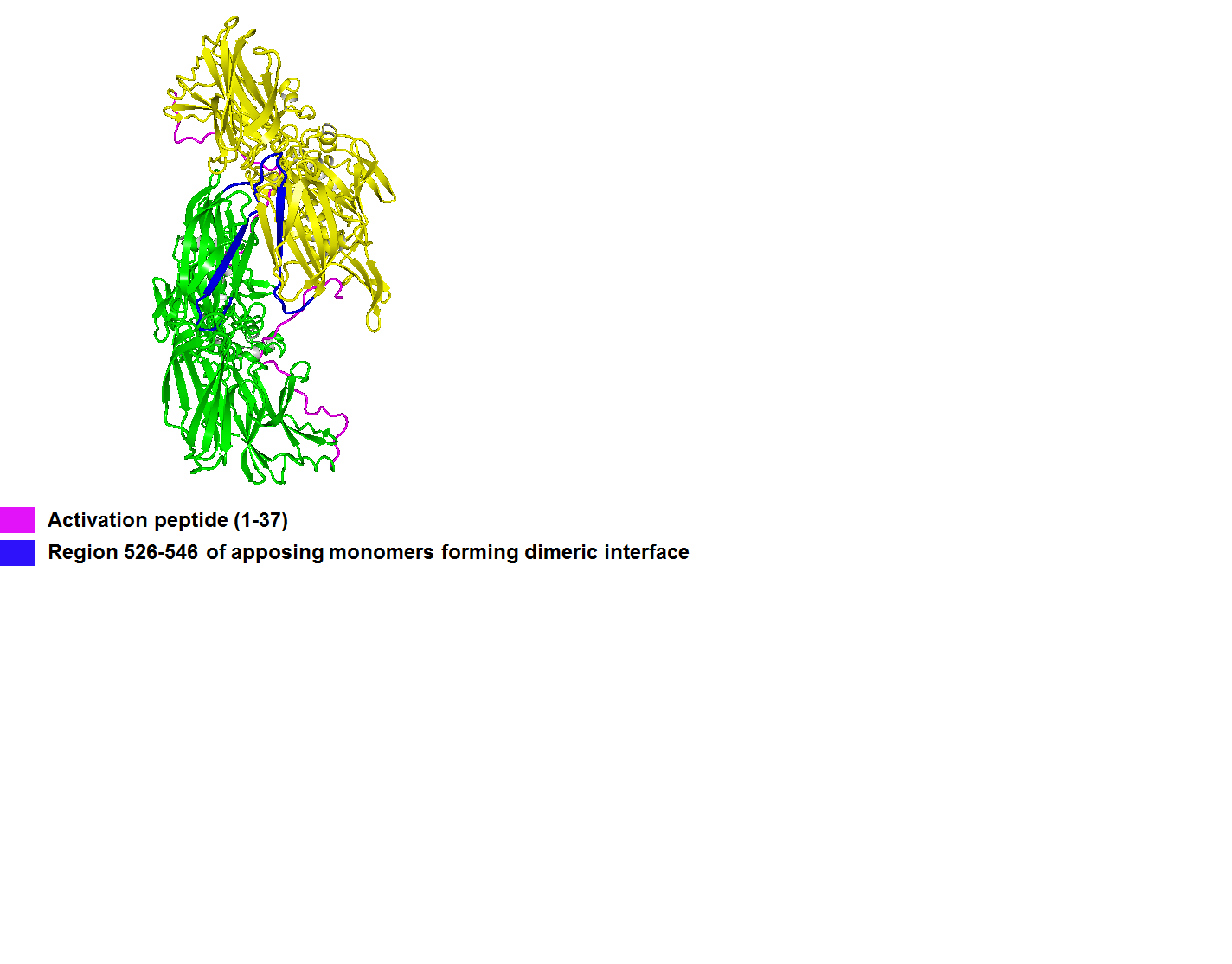
**

This model was generated by replacing the activation peptide in a monomeric chain of the FXIIIA zymogenic structure by the fully extended activation peptide resulting from SMD simulated dissociation of the FXIIIA subunit monomer from the zymogenic homodimeric form. The monomer structure was then dimerized using the MZ docking server. The residues which are retained during the FXIIIA subunit SMD simulated dissociation were used as binding constraints for the docking. The individual monomers are colored yellow and green and depicted in ribbon format. The binding interface within the barrel domains from apposing monomers are colored blue.

**Figure S12: Prediction of protease susceptibility as a comparison between the zymogenic and activated forms of FXIIIA subunit.**

**
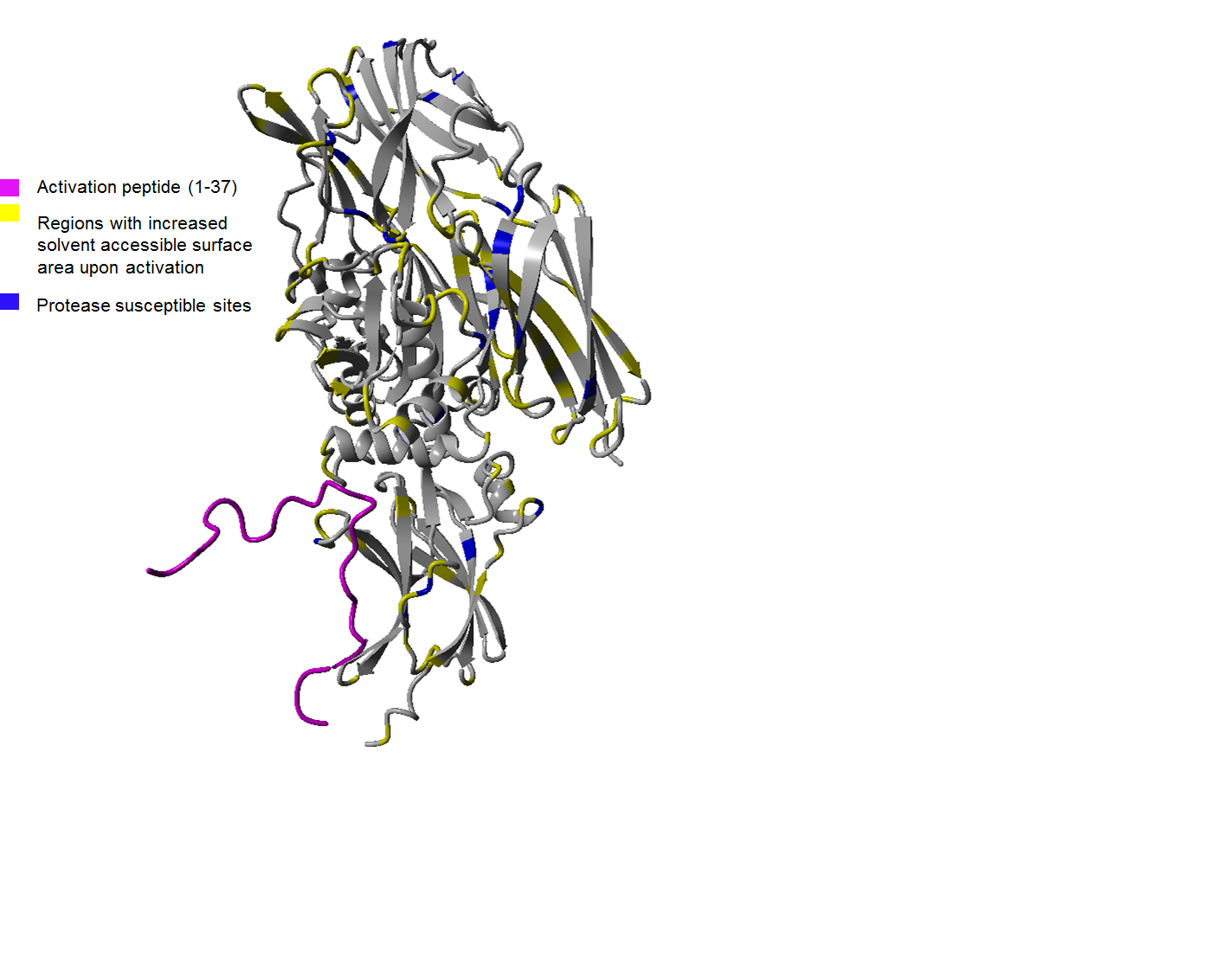
**

Shown is an alpha-carbon backbone trace (purple ribbon) for the A chain of the zymogenic FXIIIA crystal structure (PDB ID: 1f13). Computed gains (> 50Å) for solvent accessible area for activated FXIIIA residues, using the zymogen for comparison, are indicated in yellow; of the residues with increased surface accessible area, those with predicted protease specificity, as determined by Expasy server (<http://web.expasy.org/peptide_cutter/>; accessed on 11.05.2015), are colored blue.
